# On the traces of lost identities: chronological, anthropological and taphonomic analyses of the Late Neolithic/Early Eneolithic fragmented and commingled human remains from the Farneto rock shelter (Bologna, northern Italy)

**DOI:** 10.1007/s12520-023-01727-2

**Published:** 2023-03-01

**Authors:** Teresa Nicolosi, Valentina Mariotti, Sahra Talamo, Monica Miari, Laura Minarini, Gabriele Nenzioni, Fiamma Lenzi, Annalisa Pietrobelli, Rita Sorrentino, Stefano Benazzi, Maria Giovanna Belcastro

**Affiliations:** 1 Department of Biological, Geological and Environmental Sciences, Alma Mater Studiorum University of Bologna, Bologna, Italy; 2 Department of Cultural Heritage, Alma Mater Studiorum University of Bologna, Bologna, Italy; 3 Department of Chemistry ‘Giacomo Ciamician’, Alma Mater Studiorum University of Bologna, Bologna, Italy; 4 Department of Human Evolution, Max Planck Institute for Evolutionary Anthropology, Leipzig, Germany; 5 Soprintendenza Archeologia, Belle Arti e Paesaggio per la città metropolitana di Bologna e le province di Modena, Reggio Emilia e Ferrara, Bologna, Italy; 6 Museo Civico Archeologico, Bologna, Italy; 7 Museo della Preistoria ‘Luigi Donini’, San Lazzaro di Savena, Bologna, Italy

**Keywords:** Prehistory, Radiocarbon dating, Funerary practices, Corpse treatment, Perimortem lesions

## Abstract

The present study examines the prehistoric human skeletal remains retrieved starting from the 1920s in the deposit of the Farneto rock shelter, situated in the area of the ‘Parco dei Gessi Bolognesi e Calanchi dell’Abbadessa’ (San Lazzaro di Savena, Bologna, northern Italy). An exact dating and a reliable interpretation of the assemblage had not been reached so far because of the lack of contextual data useful for dating purposes, the inaccurate recovery procedures of the remains and their state of preservation. In fact, the skeletal remains from the Farneto rock shelter are highly fragmented and commingled, whereas reliable information about their original position and their recovery procedures are not available. Despite these difficulties, radiocarbon analyses allowed the precise dating of the remains to a final phase of the Neolithic and an early phase of the Eneolithic period in Emilia Romagna (northern Italy). The study of the assemblage enabled to clarify the use of the context for funerary purposes. Moreover, the anthropological and taphonomic analyses of the skeletal remains shed light on the biological profile of the individuals and on some events that occurred after their death. In particular, the analysis of perimortem lesions highlighted the existence of intentional interventions related to corpse treatment, referable to dismembering/disarticulation and scarnification, i.e. cleaning of bones from soft tissues. Finally, the comparison with other Italian and European Neo/Eneolithic funerary contexts enabled a better understanding of these complex ritual practices.

**Supplementary Information:**

The online version contains supplementary material available at 10.1007/s12520-023-01727-2.

## Introduction

In archaeological contexts, especially in prehistoric ones, it is common to find fragmented and commingled skeletal remains, both non-human and human. These assemblages can derive from deliberate anthropic intervention or accidental events of biological (e.g. animal activity, trampling) or environmental (e.g. landslides, run-off phenomena) origin. Anthropological standard approaches for the study of human remains cannot always provide a complete understanding of such contexts, making it necessary to complement them with other techniques best suited for fragmented remains, such as zooarchaeological ones (Outram et al. 2005). Thereby, it is possible to gain valuable insights into the demographic characteristics of the human group forming the assemblage, as well as to reach a better comprehension of mortuary treatments or possible funerary practices and rituals performed in those contexts (cf. Mariotti et al. 2009, 2014, 2021; Belcastro et al. 2010; Mariotti and Belcastro 2017).

### The Farneto rock shelter: discovery and retrieval of the human skeletal remains

**Fig. 1:**
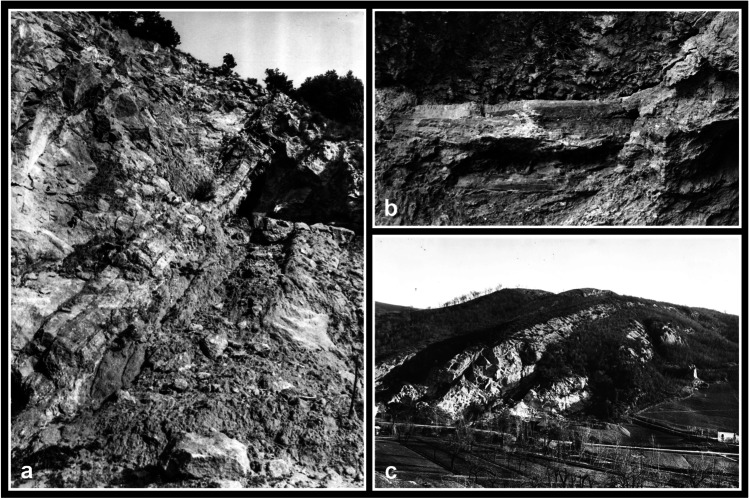
The Farneto rock shelter: **a** the deposit (ph. L. Fantini, ‘Archivio Storico di Bologna’); **b** detail of a wall of the rock shelter where it is possible to distinguish the alternation of gypsum layers (ph. L. Fantini, ‘Archivio Storico di Bologna’); **c** the hill where the Farneto rock shelter and cave are situated within the ‘Parco dei Gessi Bolognesi e Calanchi dell’Abbadessa’ (ph. L. Fantini, ‘Archivio Storico di Bologna’)

Among the prehistoric contexts of Emilia Romagna (northern Italy), the Farneto rock shelter (San Lazzaro di Savena, Bologna) represents an emblematic example of fragmented and commingled human remains assemblage. The site is a 6-m-high gypsum and clay deposit (Fig. 1a, b), situated in the central area of the ‘Parco dei Gessi Bolognesi e Calanchi dell’Abbadessa’. It was discovered in 1924 accidentally, thanks to the finding of a lithic arrowhead, by Luigi Fantini, who was neither an archaeologist nor an anthropologist, but passionate about speleology and founder of the ‘Gruppo Speleologico Bolognese’ (Busi 2018). The deposit takes the name from the nearby Farneto Cave, situated on top of a low hill (Fig. 1c), where Bronze Age artefacts and some skeletal remains had previously been retrieved by Francesco Orsoni and Edoardo Brizio (Bonometti 2018; Bonometti and Minarini 2022).

**Fig. 2:**
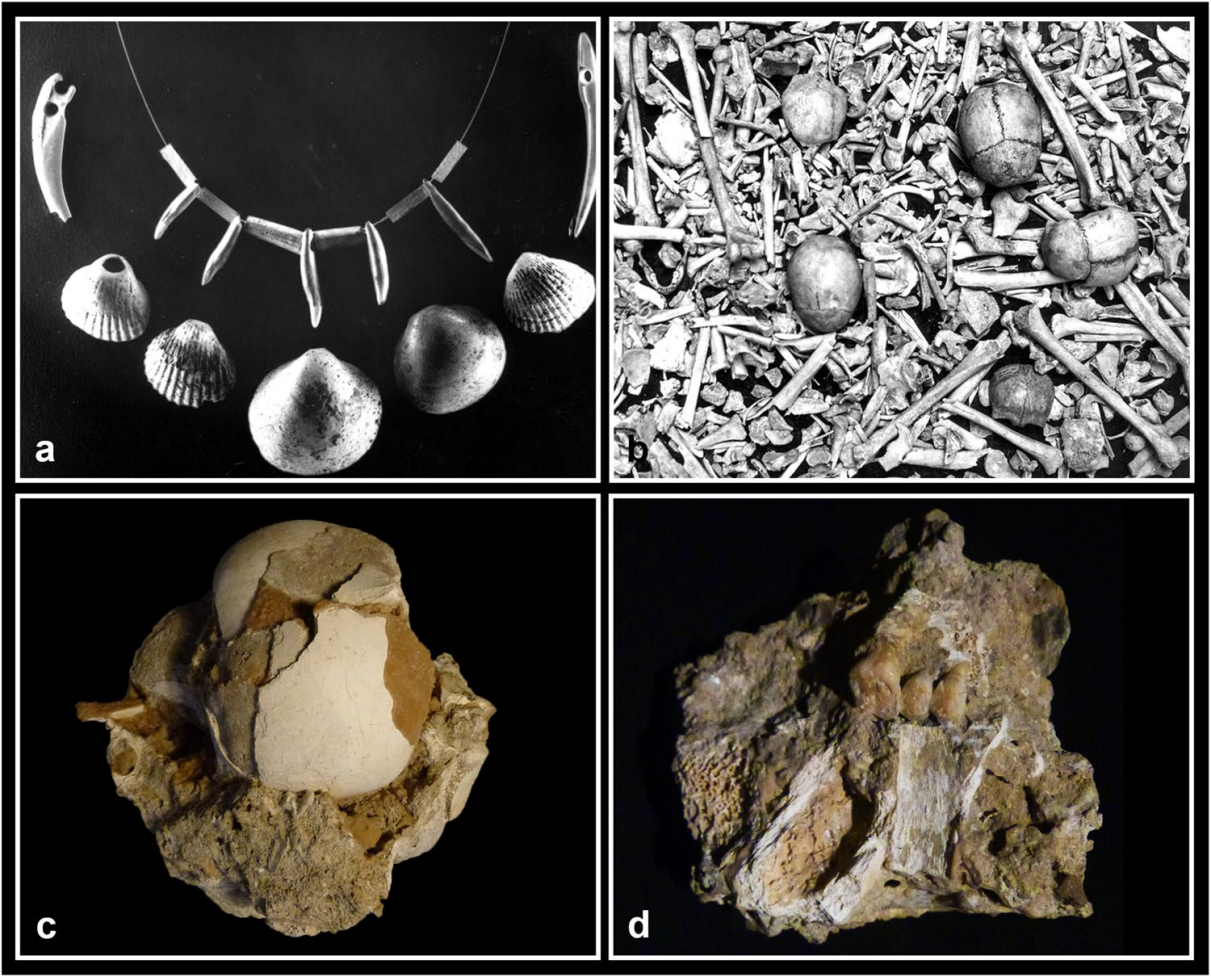
Materials retrieved at the Farneto rock shelter: **a** drilled shells and teeth interpreted as Neo/Eneolithic ornamental grave goods (ph. L. Fantini, ‘Archivio Storico di Bologna’); **b** amass of commingled human skeletal remains (ph. L. Fantini, ‘Archivio Storico di Bologna’); **c** human cranium embedded in a sediment block along with other unidentified skeletal remains and incrustations; **d** commingled teeth and bone elements embedded in a sediment block without any anatomical order

The materials retrieved at the Farneto rock shelter consist of lithic and ceramic artefacts (Bazzocchi et al. 2015; Nobili 2017), ornaments (e.g. drilled shells and teeth; Fig. 2a), antler tools (Thun Hohenstein et al. 2020), few copper objects (a probable ornament and metal waste) and a large amount of skeletal remains, mostly human (Fig. 2b). L. Fantini went periodically to the rock shelter from 1924 to 1970, noticing that new objects and fragmented skeletal remains came to light in the deposit after every heavy rain and landslide (Busi 2018). In particular, in 1969–1970, the activities of a nearby gypsum quarry brought to the collapse of some parts of the deposit allowing the collection of many bone fragments, mostly embedded in a chalky soil (Facchini 1972; Fig. 2c, d).

According to L. Fantini’s written descriptions, most of the skeletal remains were already fragmented and chaotically placed at the time of their retrieval, except for a probable partial primary burial detected in 1954 that was not excavated because it was embedded in a stalagmitic crust (Fantini 1959, 1969; Facchini 1972; Busi 2018). Unfortunately, we do not have any clear photographic or graphic documentation regarding the original disposition and placement of the skeletal remains. The fragmentation and stratification of the skeletal remains in the deposit made L. Fantini think that they could have originated from a higher place, probably a cave or a natural cavity used for funerary purposes, then slipped down due to natural mudslides and rockfalls that took place in ancient times and in most recent years (Fantini 1959, 1969). This hypothesis is still possible because of the geomorphological conformation of the area in the northern Apennines, where a high-frequency alternation of cave formation and sedimentation periods has been documented in many gypsum caves (Pisani et al. 2019). On the other hand, soil movements were also influenced by the activity of the nearby gypsum quarry, where mineral was extracted through the use of explosives (Facchini 1971, 1972; Busi 2018).

### Proposed chronology and study of the materials

Related to the still open questions of the original position of the Farneto archaeological and anthropological findings are the doubts about the dating of the context. After the discovery, L. Fantini supposed that the materials were datable to the Neolithic/Early Eneolithic period, on the basis of lithic and pottery typologies (Fantini 1959). Other archaeologists and speleologists proposed different dates, in particular, the last phase of the Eneolithic/Early Bronze Age (Scarani 1964) or the Ancient Bronze Age, coeval with the occupation of the abovementioned Farneto Cave (Malavolti 1948). In the absence of absolute radiocarbon dating, L. Fantini’s hypothesis has been the most widely shared by the scientific community on the basis of some archaeological artefacts (Bazzocchi et al. 2015; Nobili 2017; Nenzioni and Lenzi 2022).

During the long period of the discoveries, the findings were divided among different institutions, i.e. the ‘Museo Civico Archeologico’ of Bologna and the former Institute of Anthropology of the University of Bologna. In addition, today a small batch of materials is stored at the ‘Museo della Preistoria Luigi Donini’ in San Lazzaro di Savena (Bologna; S1). The skeletal remains from the Farneto rock shelter were firstly studied by Fabio Frassetto (1939), but especially during the 1960s and the early 1970s by Fiorenzo Facchini, to gain information on the biological and metric features of the individuals (Facchini 1962, 1970, 1971, 1972). In the last few years, interest for these materials has grown again (Romagnoli 2018; Nicolosi 2019), partly due to the recovery of an Early Eneolithic isolated cranium inside the nearby Marcel Loubens Cave (San Lazzaro di Savena, Bologna; Belcastro et al. 2021). The restudy of the skeletal remains from the Farneto rock shelter may represent an important opportunity to shed light on many aspects of the prehistoric period in northern Italy, and in Emilia Romagna in particular, especially regarding the reconstruction of funerary practices inside natural cavities (Miari 2013, 2018).

In 2018, we began the restudy of the human skeletal remains of the Farneto rock shelter (preliminary results in Miari et al. 2022) with two goals:

1. To clarify the chronological range to which the skeletal remains can be ascribed through radiometric dating (^14^C);
2. To shed light on the origin of the assemblage through the analysis of possible traces of deliberate human intervention on the bones (perimortem vs. postmortem fractures, traces of treatment of the cadaver; cf. Mariotti et al. 2009, 2020, 2021; Belcastro et al. 2010, 2021).

The aim of this work is to show the results of our chronological and anthropological study. We also hope to achieve a better comprehension of the funerary behaviour of the Neo/Eneolithic populations of northern Italy by comparing our findings with those of other studies on coeval funerary contexts.

## Materials and methods

### The human skeletal remains assemblage

The fragmented and commingled human remains from the Farneto rock shelter are today kept in three different institutions: the ‘Museo Civico Archeologico’ of Bologna (MCA), the ‘Collezioni di Antropologia’ of the Museum System of the University of Bologna (CA) and the ‘Museo della Preistoria Luigi Donini’ in San Lazzaro di Savena (MPLD; S1). All necessary permits for the present study were obtained by the ‘Soprintendenza Archeologia, Belle Arti e Paesaggio per la città metropolitana di Bologna e le province di Modena, Reggio Emilia e Ferrara’ (SABAP-BO) and granted by one of the authors, Monica Miari, referent for the abovementioned Institution.

In the three museums, the remains from the Farneto rock shelter are only partially displayed in showcases, while most of them are stored in numbered boxes and bags of different dimensions. Different types of bone are commingled, without any apparent order and coherence.

Besides the splitting of the materials, the study of the assemblage is further complicated by the possible mixing with the skeletal remains from the Farneto Cave, that belong to three individuals (an adult female: cranium, mandible, right humeral diaphysis, right ilium, some vertebrae, costal fragments; an adult male: mandible, petrous portions of the temporal bones, some vertebrae, right ilium, a femoral fragment, a fibula, some ribs; a child: maxillary and mandibular fragments; Frassetto 1905). During the last century, the few skeletal remains from the Farneto Cave were originally displayed in the showcases and then stored in the magazines of the MCA (Bonometti and Minarini 2022). Today, they are no longer identifiable and have likely been mixed with the skeletal remains from the Farneto rock shelter. Only two partially preserved vertebrae stored at the CA are clearly indicated as originating from the Cave, along with plaster casts of the adult female skull (cranium and mandible) and the adult male mandible. Thanks to these casts, it was possible to recognise the female original maxilla and mandible among the materials stored at the MCA, that were so excluded from the present study, while the male mandible is lost.

### Radiometric analyses

Bone and dental elements from the CA were given to the Department of Human Evolution at the Max Planck Institute for Evolutionary Anthropology (MPI-EVA, Leipzig, Germany) to perform radiometric analyses (^14^C). At first, a tooth and three diaphyseal fragments were delivered in 2018. Then, another 15 specimens (mandibular fragments or teeth) belonging to different individuals were also subjected to analysis between 2019 and 2020.

All samples were pretreated using the method described in Talamo et al. (2021). The outer surface of the bone samples is first cleaned by a shot blaster and then 500 mg of the whole bone is taken. The samples are then decalcified in 0.5 M HCl at room temperature until no CO_2_ effervescence is observed. 0.1 M NaOH is added for 30 min to remove humics. The NaOH step is followed by a final 0.5 M HCl step for 15 min. The resulting solid is gelatinised following Longin (1971) at pH3 in a heater block at 75 °C for 20 h. The gelatine is then filtered in an Ezee-Filter™ (Elkay Laboratory Products (UK) Ltd.) to remove small (> 80 μm) particles. The gelatine is then ultrafiltered (Brown et al. 1988; Talamo et al. 2021) with Sartorius ‘VivaspinTurbo’ 30-KDa ultrafilters. Prior to use, the filter is cleaned to remove carbon-containing humectants (Talamo et al. 2021). The samples are lyophilised for 48 h. All dates were corrected for a residual preparation background estimated from ^14^C free bone samples. These bones were kindly provided by the Mannheim laboratory and pretreated in the same way as the archaeological samples (Korlević et al. 2018). Between 3 and 5 mg of collagen were inserted into pre-cleaned tin capsules. These were sent to the Mannheim AMS laboratory (Lab Code MAMS) where they were graphitised and dated (Kromer et al. 2013).

In parallel, two bone samples from the MPLD were delivered in 2018 at CEDAD (‘Centro di Datazione e Diagnostica dell’Università del Salento’) to obtain absolute dates. Radiocarbon concentration was determined by comparing measured values of ^12^C and ^13^C currents and ^14^C counts with values obtained from standard samples of C6 sucrose provided by the IAEA. Conventional radiocarbon dating was corrected for isotope fractionation effects both by measuring the δ^13^C term directly with the accelerator and by the background of the measurement. Samples of known concentration of oxalic acid provided by the NIST (National Institute of Standards and Technology) were used as a quality control for the results. Both the scattering of the data around the mean value and the statistical error from counting ^14^C were taken into account in determining the experimental error in the radiocarbon date.

All obtained ^14^C dates were calibrated using the IntCal20 curve within the OxCal 4.4 program (Bronk Ramsey 2009; Reimer et al. 2020).

### Anthropological and taphonomic analyses

Firstly, we made a new complete inventory of the overall assemblage from the Farneto rock shelter housed in the three institutions. The bone fragments embedded inside sediments were not counted, because they are not completely distinguishable, with the exception of a calotte partially embedded and thus clearly visible (Fig. 2c). No bones were restored, in order to preserve the original state of fragmentation and study the characteristics of fracture margins. However, some skeletal remains, especially crania, had been restored during previous studies. Each restored element was counted as one in the inventory.

The inventory contains the following information: previous number of inventory, type of bone and side, state of preservation, maximum length, sex and age class, colour, eventual presence of burning and gnawing traces, possible perimortem lesions.

The state of preservation was recorded as follows: 1 complete, 2 almost complete, 3 diaphysis + proximal epiphysis, 4 diaphysis + distal epiphysis, 5 fragmented (5.1 fragment of cancellous bone, 5.2 fragment of cortical bone, 5.3 fragment of both cancellous and cortical bone; cf. Outram 2001), 6 unfused proximal epiphysis, 7 unfused distal epiphysis. Codes 1, 2 and 5 may refer to all skeletal districts, while codes 3, 4, 6 and 7 clearly refer to long bones.

The maximum length of each fragment was measured with a sliding calliper (sensitivity: 1 mm) to study the degree of fragmentation and the most attested length classes (Outram 2001).

The minimum number of elements (MNE) was calculated according to Knüsel and Outram (2006), additionally distinguishing atlas and axis from the rest of cervical vertebrae, ilium, ischium and pubis from *os coxae*, each carpal and metacarpal and each tarsal and metatarsal. Adult vs. subadult elements and left vs. right sides were treated separately. Thanks to that, the minimum number of individuals (MNI) was calculated. Then, element representation index (ERI) was calculated, dividing the observed number of each element by the number of elements expected assuming that each individual (considering the MNI) was represented by a complete skeleton (cf. Robb et al. 2015).

For the determination of sex, we referred to current morphological methods (Ferembach et al. 1980; Loth and Henneberg 1996) and, where possible, we measured the diameter of the femoral head (Bass 2001).

For the estimation of adult age at death, we used occlusal dental wear methods (Brothwell 1981; Lovejoy 1985) and we looked at the persistence of the epiphyseal line in the appendicular skeleton (Belcastro et al. 2019). As regards subadults, we observed the formation and eruption of deciduous and permanent teeth (Mincer et al. 1993; AlQahtani et al. 2010) and we measured the maximum length of the diaphysis without epiphyses (Schaefer et al. 2009). For adolescents, we observed the epiphyseal fusion degree (Schaefer et al. 2009). We then considered the following age classes (Buikstra and Ubelaker 1994): infant (IN, birth–3 years), child (CH, 4–12 years), adolescent (ADOL, 13–20 years), young adult (YA, 21–35 years), mature or middle adult (MA, 36–50 years), old adult (OA, > 50 years).

Taphonomic changes were recorded for each bone, even fragmented or incomplete. For the colour of bones, a standard was created on the basis of the nuances of the bones of the sample: 1.1 very light whitish, 1.2 very light reddish, 2 brown (cf. Dupras and Schultz 2013). The presence of burning traces, represented by colour changes, reduction, warping, cracking and fracturing, was assessed referring to the relevant literature (Shipman et al. 1984; de Becqdelievre et al. 2015). Evidence of rodent and carnivore gnawing activity was recorded according to Pokines (2013) and Knüsel and Robb (2016). Rodent incisors leave on bone paired, broad, shallow and flat-bottomed grooves (Knüsel and Robb 2016). As regards carnivore tooth marks, the authors distinguish four types: tooth pits, punctures, scores and furrows. Tooth pits consist of circular to irregular-shaped depressions in the cortical bone, which do not penetrate the bone interior, while punctures (or perforations; Andrews and Fernández-Jalvo 2012) are deeper depressions that penetrate the interior of the bone (Pokines 2013).

Fracture patterns were studied to assess if fracturing was produced on fresh, dry (i.e. bone free of soft tissue, but maintaining a certain quantity of organic components) or mineralised bone. For long bones, the observed characteristics include fracture angle, surface texture and outline. In particular, a fresh bone fracture may present a notional 10% of the fracture surface perpendicular to the cortical one, its surface is smooth, while the fracture outline is mostly helical. Mineralised fractures present a straight outline and a largely rough surface, mostly perpendicular to the bone surface. Dry fractures display mixed features (Villa and Mahieu 1991; Outram 2001, 2002). For cranial fractures and other specific fracture patterns, we referred to the relevant literature (e.g. Wedel and Galloway 2014).

Finally, possible sharp force lesions (such as cut marks, chop marks and perforating lesions; Kimmerle and Baraybar 2008) were investigated macroscopically and under a stereomicroscope. We noted their type, position and characteristics to determine their timing (ante-, peri- or postmortem) and the action that may have caused them (White 1992; Olsen and Shipman 1994; Blumenschine et al. 1996; White and Folkens 2005; Domínguez-Rodrigo et al. 2009; Andrews and Fernández-Jalvo 2012; cf. Mariotti et al. 2020).

The particular karstic environment of the Farneto rock shelter, its use as a quarry and the mode of recovery of the bones could account for most of the fragmentation and bad preservation of the skeletal material, sometimes still included in sediments or covered by incrustations. For this reason, we did not calculate the frequency of lesions (gnawing traces, fractures, sharp force lesions) and the most affected skeletal districts. In addition, results regarding the type of fractures will be given only for those specimens presenting any kind of perimortem lesions.

## Results

### Radiometric dates

Among the 20 specimens for which radiometric dates were obtained by ^14^C analyses, 13 samples from the CA and two from the MPLD are attributable to the first half of the IV millennium BC, while only five support a completely different chronology (Table 1). These latter samples present a brownish colour that is very different from the white colour of all the other materials. In light of these results, we excluded from the prehistoric inventory of the Farneto rock shelter all specimens with brown colour (i.e. the abovementioned five samples, two other elements from the CA, one from the MCA and two others from the MPLD). In addition, among the specimens of the MPLD, the complete skull of an approximately 4-year-old child was impossible to date through radiocarbon analysis due to the lack of organic substance. This skull was particularly well preserved, in contrast to the rest of the assemblage, so it was also excluded from further analyses.

**Table 1:** Results of the radiometric dates obtained by ^14^C analyses with the indication of sample name, submitter code, collagen mass (in mg), percentage of collagen, AMS Code, results Before Present (BP), 1-sigma and 2-sigma probability, collection, type and colour of samples. The ^14^C dates were calibrated using the IntCal20 curve within the OxCal 4.4 program (Bronk Ramsey 2009; Reimer et al. 2020). Colour codes follow the standard described in the ‘Methods’ section

Sample name	Submitter code	Collagen mass (mg)	%Coll	AMS Code	^14^C Age [yr BP]	±	Cal BC/AD 1-sigma	Cal BC/AD 2-sigma	Collection	Type	Colour
R-EVA 3416	V-AMH-200.B	80.2	14.31	MAMS-48094	4998	21	3796–3711	3933–3659	CA	Tooth (mandible)	1.1
R-EVA 3417	V-AMH-202	26.3	7.3	MAMS-48095	4954	21	3765–3655	3777–3652	CA	Tooth (mandible)	1.1
R-EVA 3426	C3-AMH-261.B	31.4	7.66	MAMS-48104	4936	21	3710–3651	3768–3648	CA	Isolated tooth	-
R-EVA 3425	C3-AMH-261.A	89	13.95	MAMS-48103	4934	21	3709–3652	3768–3647	CA	Isolated tooth	-
R-EVA 3415	C1-AMH-267	66	13.05	MAMS-48093	4933	21	3709–3651	3768–3647	CA	Tooth (mandible)	1.2
R-EVA 3422	C4-AMH-297.1	64.4	13.44	MAMS-48100	4932	21	3709–3651	3768–3647	CA	Tooth (mandible)	1.1
R-EVA 3421	C4-AMH-297.2	78.2	13.09	MAMS-48099	4931	21	3708–3651	3768–3646	CA	Tooth (mandible)	1.1
R-EVA 3135	C4-AMH-261	37.9	7.4	MAMS-42295	4911	22	3704–3647	3759–3640	CA	Isolated tooth	-
R-EVA 3419	C4-AMH-298	64.7	10.5	MAMS-48097	4905	22	3703–3645	3754–3638	CA	Tooth (mandible)	1.1
R-EVA 3427	C4-AMH-297.A	55.1	12.59	MAMS-48105	4886	21	3699–3640	3708–3636	CA	Isolated tooth	-
R-EVA 3137	C4-AMH-298	99.5	14.8	MAMS-42297	4840	22	3646–3541	3652–3532	CA	Diaphysis fragment	1.1
R-EVA 3139	C4 AMH-298	62.5	10	MAMS-42301	4830	22	3644–3539	3650–3531	CA	Diaphysis fragment	1.1
R-EVA 3136	C4-AMH-298	25.3	4.9	MAMS-42296	4757	21	3626–3526	3634–3386	CA	Diaphysis fragment	1.1
R-EVA 3428	C4-518	17.5	6.69	MAMS-48106	202	17	1660–1799	1655–…	CA	Mandible fragment	2
R-EVA 3420	C4-AMH-288	51.6	15.44	MAMS-48098	201	17	1661–1799	1656–…	CA	Tooth (mandible)	2
R-EVA 3418	C4-AMH-289	74.9	15.07	MAMS-48096	147	17	1681–1940	1671–1945	CA	Mandible fragment	2
R-EVA 3423	C4-AMH-287	39.3	7.76	MAMS-48101	106	17	1696–1915	1693–1919	CA	Tooth (mandible)	2
R-EVA 3424	C4-AMH-519	84.5	15.91	MAMS-48102	2461	19	749–516	755–421	CA	Tooth (mandible)	2
LTL 18584A	INV-6778	-	-	-	4858	45	3705–3535	3761–3526	MPLD	Diaphysis fragment	1.1
LTL 18585A	-	-	-	-	4793	45	3637–3529	3649–3382	MPLD	Diaphysis fragment	1.1

Abbreviations: *%Coll* percentage of collagen, *yr BP* years Before Present, *CA* ‘Collezioni di Antropologia’, *MPLD* ‘Museo della Preistoria Luigi Donini’

### State of preservation, MNE, MNI, ERI and biological profile of the individuals

Table 1 shows the collagen content of the specimens used for radiocarbon dating. The five bone fragments give a mean value of collagen content equal to 10.3%, while considering the three prehistoric and two historic fragments separately the values are 9.9% and 10.9% respectively.

The inventory of the prehistoric skeletal remains from the Farneto rock shelter includes 2622 elements (single bone fragments, previously restored bones and isolated teeth; Table 2). The great majority of elements belongs to the MCA and CA, while the MPLD hosts only 38 elements. We identified 2069 elements (78.9%) at least for the bone type, while 553 (382 fragments of cortical bone/diaphysis + 171 fragments of cancellous or mixed bone: 21.1%) remain unidentified.

**Table 2:** Number of fragmented and complete/almost complete elements per skeletal district

District	Fragments*	Complete/almost complete elements (even if restored)*	Complete/almost complete subadults unfused elements	TOT
Isolated teeth	36	115	-	151
Cranium/calotte	508	16	2	526
Mandible	36	4	-	40
Maxilla	27	0	-	27
Hyoid	1	0	-	1
Vertebrae	103	23	2	128
Sacrum and coccyx	7	2	4	13
Sternum	2	0	-	2
Ribs	378	9	-	387
Clavicle	21	3	-	24
Scapula	37	1	-	38
Humerus	64	3	2	69
Radius	42	2	0	44
Ulna	41	2	0	43
Carpals	1	5	-	6
Metacarpals	25	22	0	47
Hand phalanges	14	50	2	66
Os coxae	48	1	10	59
Femur	100	5	6	111
Patella	2	10	-	12
Tibia	67	5	2	74
Fibula	63	0	0	63
Talus	1	18	-	19
Calcaneus	11	7	2	20
Cuboid	0	3	-	3
Navicular	0	5	-	5
Cuneiforms	2	6	-	8
Metatarsals	32	26	0	58
Foot phalanges	3	22	0	25
Unidentified cortical bone/diaphysis	382	0	-	382
Unidentified cancellous or mixed bone	171	0	-	171
**TOT**	2225	365	32	2622

^*^Adult and subadult specimens are considered together because they are often indistinguishable. When unfused elements are observable, they are indicated in the ‘complete/almost complete subadults unfused elements’ column

**Fig. 3:**
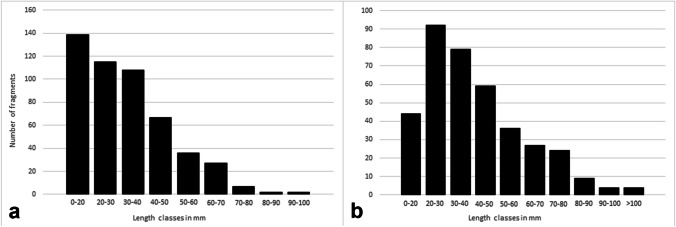
Distribution of fragments by length classes (in mm): **a** cranial fragments (crania restored during previous studies are not considered); **b** unidentified long bone diaphysis fragments

The distribution of fragments (adult and subadult together) by length classes for crania (mandibles excluded) and unidentified long bones is shown in Fig. 3. For the 508 cranial fragments, 71.3% measure less than 40 mm, while 84.4% measure less than 50 mm (Fig. 3a). For the 382 long bone fragments, 71.7% measure less than 50 mm (Fig. 3b). Among the identified adult long bones, the commonest class is > 100 mm.

Detailed results regarding the MNE based on identified elements, the MNI for adults and subadults, the ERI of each bone, distinguishing adults vs. subadults and left vs. right sides, are shown in Table 3.

**Table 3:** Results of the MNE, MNI and ERI calculated for each bone district on the basis of the identified elements for adults and subadults, left vs. right sides and unpaired elements separately

	MNE				MNI		ERI (%)			
	AD		SUB		AD	SUB	AD (MNI = 14)		SUB (MNI = 10)*	
District	left	right	left	right			left	right	left	right
Calotte	14		3		**14**	3	100.0		30.0	
Mandible	10		7		10	7	71.4		70.0	
Maxilla	6	4	8	8	6	8	42.9	28.6	80.0	80.0
Hyoid	1		0		1	0	7.1		0	
Atlas	3		0		3	0	21.4		0	
Axis	4		0		4	0	28.6		0	
Cervical vertebrae	8		2		2	1	11.4		4.0	
Thoracic vertebrae	26		7		3	1	15.5		5.8	
Lumbar vertebrae	9		8		2	2	12.9		16.0	
Sacrum	5		5		5	5	35.7		50.0	
Sternum	2		0		2	0	14.3		0	
Clavicle	5	4	4	4	5	4	35.7	28.6	40.0	40.0
Scapula	5	3	4	7	5	7	35.7	21.4	40.0	70.0
Humerus	7	12	6	4	12	6	50.0	85.7	60.0	40.0
Radius	9	7	2	2	9	2	64.3	50.0	20.0	20.0
Ulna	12	6	2	7	12	7	85.7	42.9	20.0	70.0
Scaphoid	0	0	0	0	0	0	0	0	0	0
Lunate	0	0	0	0	0	0	0	0	0	0
Triquetral	0	0	0	0	0	0	0	0	0	0
Pisiform	0	0	0	0	0	0	0	0	0	0
Trapezium	2	0	0	0	2	0	14.3	0	0	0
Trapezoid	1	0	0	0	1	0	7.1	0	0	0
Capitate	1	1	0	0	1	0	7.1	7.1	0	0
Hamate	1	0	0	0	1	0	7.1	0	0	0
I metacarpal	1	3	0	0	3	0	7.1	21.4	0	0
II metacarpal	4	3	0	0	4	0	28.6	21.4	0	0
III metacarpal	3	1	0	1	3	1	21.4	7.1	0	10.0
IV metacarpal	4	2	0	0	4	0	28.6	14.3	0	0
V metacarpal	3	6	1	2	6	2	21.4	42.9	10.0	20.0
Hand phalanges	42		21		2	1	10.7		7.5	
Ilium	1	6	3	9	6	**9**	7.1	42.9	30.0	90.0
Ischium	1	2	7	1	2	7	7.1	14.3	70.0	10.0
Pubis	1	0	5	2	1	5	7.1	0	50.0	20.0
Femur	10	12	6	4	12	6	71.4	85.7	60.0	40.0
Patella	5	5	1	1	5	1	35.7	35.7	10.0	10.0
Tibia	10	4	6	6	10	6	71.4	28.6	60.0	60.0
Fibula	8	4	2	0	8	2	57.1	28.6	20.0	0.0
Talus	7	6	3	2	7	3	50.0	42.9	30.0	20.0
Calcaneus	4	4	1	1	4	1	28.6	28.6	10.0	10.0
Cuboid	2	1	0	0	2	0	14.3	7.1	0	0
Navicular	2	2	0	1	2	1	14.3	14.3	0	10.0
I cuneiform	3	1	0	0	3	0	21.4	7.1	0	0
II cuneiform	1	1	0	0	1	0	7.1	7.1	0	0
III cuneiform	0	0	0	0	0	0	0	0	0	0
I metatarsal	6	2	3	1	6	3	42.9	14.3	30.0	10.0
II metatarsal	4	2	0	1	4	1	28.6	14.3	0	10.0
III metatarsal	2	2	1	0	2	1	14.3	14.3	10.0	0
IV metatarsal	2	4	2	3	4	3	14.3	28.6	20.0	30.0
V metatarsal	5	2	1	2	5	2	35.7	14.3	10.0	20.0
Foot phalanges	20		4		2	1	5.1		1.4	
TOT	138	112	68	69						

The MNI is indicated in bold

Abbreviations: *MNE* minimum number of elements, *MNI* minimum number of individuals, *AD* adults, *SUB* subadults, *ERI (%)* element representation index

^*^For subadult individuals, the MNI is equal to 10 because of an infant complete left humerus (C4-61), which is much younger (around 6-month-old) compared to all other infant bones

The overall MNI, considering the commonest skeletal elements and all possible associations on the basis of the relative age classes, is 24. On the basis of calottes, a total of 14 adults were recognised (Table 3), among which six males and three females. On the basis of the measurement of the diameter of the preserved femoral heads, two males and six females were recognised. Thus, at least six males and six females are present within the assemblage.

As regards subadults, nine individuals can be attributable to the classes of infant and child because of nine right ilium bones (Table 3). A younger infant around 6-month-old is represented only by a complete left humerus (C4-61) and should be added to the subadults, increasing their total number to 10. At least two adolescents are also present, represented by two maxillary bones with third molars in formation (C3-AMH-259 and C2-12) and possibly by other partially fused bones (e.g. the right tibia C7-AMH-223). Since their calottes cannot be distinguished from those of the young adults, their presence does not alter the MNI already inferred from cranial remains of adult individuals (always lacking the spheno-occipital synchondrosis). Due to this eventuality, although 12 subadult individuals should be present, we decided to keep the MNI equal to 24 (14 adults: 58.3%; 10 subadults: 41.7%).

### Bone colour, burning and gnawing traces

#### Colour

Most elements (2471: 94.2%) have white colour, while only 151 elements (5.8%), mostly from subadults, have a light reddish surface.

#### Burning

**Fig. 4:**
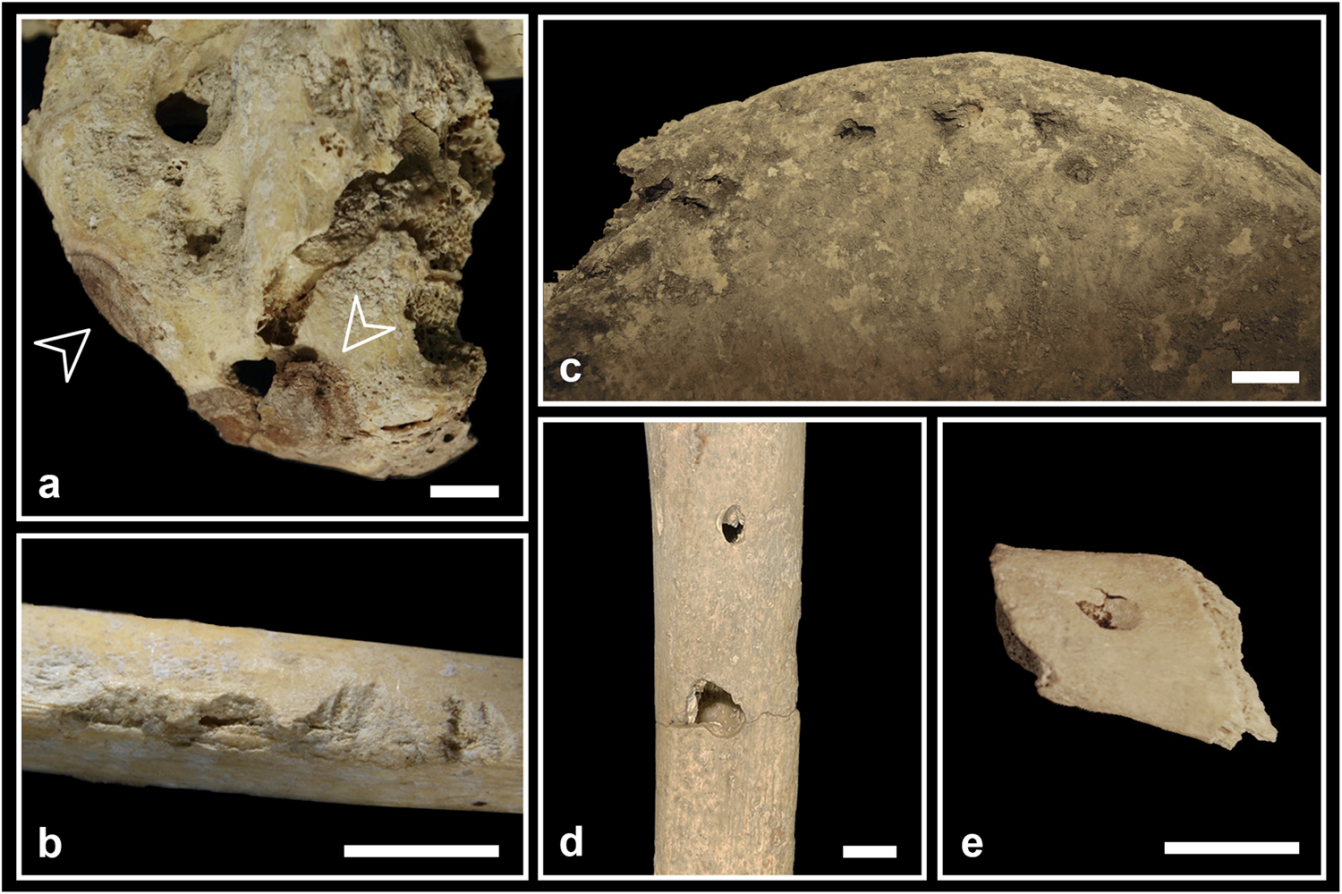
Taphonomic bone modifications: **a** the almost complete sacrum C3-AMH-264 showing traces of burning with circumscribed reddish brown discolorations indicated by the arrows (posterior view); **b** the subadult fibula C2-277 showing rodent gnawing traces along the mid-diaphysis consisting of paired and shallow grooves (ph. stereomicroscope); **c** the right ilium C5-BL2-101 affected by several pits in correspondence to the crest (ventral view); **d** the complete adolescent right tibia C7-AMH-223 affected by two puncture marks at a distance of 38 mm on the lateral diaphysis; **e** the costal fragment C8-279 affected by a puncture mark near the sternal end (external surface). Bar = 10 mm

The effect of fire can be hypothesised on only six elements (0.2%): five cranial fragments (two identified as C2-AMH-282 and three as C5-BL3-SN) and an almost complete sacrum (C3-AMH-264; Fig. 4a) show reddish brown circumscribed stains (less than 20 mm in diameter). No reduction, warping, cracking or fracturing are observable.

#### Gnawing

Traces attributable to rodents and carnivores were found on a few postcranial elements (Fig. 4b, c, d, e). In some cases, these traces are doubtful because of the bad preservation of the cortical surface. Two bones bear quite clear signs of rodent teeth (the adult right tibia C7-227 on various parts of the diaphysis and the subadult fibula C2-277 at about mid-diaphysis; Fig. 4b). Evidence of possible carnivore gnawing, testified by pits and punctures, has been found in at least six postcranial bones. The complete left femur V-AMH-211 shows a pit (4 mm) on the posterior part of the epiphyseal line of the head. The complete left femur V-AMH-212 shows two pits (3 mm) at a distance of 5 mm and a crushed lesion (10 × 6 mm) on the femoral head. The right ilium C5-BL2-101 shows many pits (about 5 mm) near the crest on both surfaces of the bone (Fig. 4c). The complete adolescent right tibia C7-AMH-223 shows two puncture marks, both perforating the lateral surface of the diaphysis, at a distance of 38 mm from the centre of the holes (Fig. 4d). The proximal lesion has a regular elliptical shape (8 × 4 mm), while the distal one presents a more irregular shape (13 × 9 mm). The almost complete (missing only the distal end) right ulna C4-298 presents a puncture (12 × 6 mm) on the posterior surface of the olecranon. The costal fragment C8-279 displays a puncture (7 × 4 mm) near the sternal end, on the external surface (Fig. 4e).

### Perimortem lesions

Lesions compatible with intentional human perimortem interventions (sharp force trauma, blunt force trauma and fractures) are described in Table 4 for skulls and S2 for postcranial bones. As regards skulls, these lesions are detectable on five adult calottes (three male and two female), a frontal bone of a child and three mandibles (two adult male and one child). Sharp force lesions affect the five calottes, the child frontal bone and one adult mandible. Cranial fractures due to blunt force trauma have been found on four calottes (two male and two female), while the child mandible is fractured on the left part of the corpus. Incomplete fractures have been detected on the inner surface of the ramus of the two adult mandibles (Table 4).

**Table 4:** Perimortem lesions detected on cranial bones with the indication of ID, district, sex and age of the individual, type and position of the lesion, description and interpretation distinguished for sharp force and blunt force trauma

			Sharp force lesions				Blunt force lesions/fractures	
ID	District	Sex, age	Type	Position	Description	Interpretation	Description	Interpretation
V-2	Calotte	F, AD	Chop mark?	Left frontal squama	Chop (?) mark (11 × 4 mm) transversally oriented, wide but not deep	Lesions are difficult to detect and interpret because the calotte is completely covered by a protective coat of consolidant	Semi-circular depressed fracture (around 42 × 19 mm) between the left frontal and parietal bones Lacuna of irregular shape (32 × 16 mm) on the right parietal bone with radiating fractures; external margins present some semi-circular flaking (covered by consolidant) as if they were retouched. Anteriorly, two radiating lines departing from the lacuna delimit a depressed fracture	Patterned injury, possibly due to blunt force trauma The lacuna could be the result of one or two strokes performed with a sharp or blunt object that caused the perforation of the surface of the bone and the related radiating fractures May flaking be related to therapeutic interventions on the wound? Interpersonal or ritual violence?
V-2B	Calotte	M, AD	Cut mark	Right frontal	Cut mark (3 mm) transversally oriented, with flaked margins	Defleshing of the cranium by severing the epicranius, perhaps related to the detachment of the mandible	Incomplete and slightly depressed fracture on the left frontal bone (around 20 mm) Several incomplete fractures departing from a large postmortem breakage on the left parietal bone; the anterior portion of the breakage shows a thin area of superficial decortication	Interpretation is very difficult because of extensive postmortem breakages
			Chop mark	Right supramastoid crest	V-shaped chop mark (4 mm) on the temporalis muscle enthesis, with the posterior margin more vertically oriented; both margins present flaking			
V-3	Calotte	M, AD	Chop mark	Left frontal	Oblique chop mark (8 × 2 mm) on the frontal bone; the antero-medial margin is vertical and straight, while the other one is more oblique and irregular (Fig. 5a)	The protective resin covering the vault obliterates the lesions making them difficult to interpret The size of the chop mark on the parietal and the perforating lesion on the temporal bone suggest violent actions (interpersonal or ritual violence?) May the cut marks be related to therapeutic interventions?		
			Chop mark?	Left parietal	Another chop (?) mark (21 × 3 mm) on the parietal bone, with flat bottom (covered by resin) and vertical inferior margin; superiorly a flake (8 × 3 mm) has been removed (Fig. 5b)			
			Perforating lesion and cut marks	Left temporal	Three cut marks (maximum length 16 mm) cross perpendicularly a perforating lesion (13 × 2 mm; Fig. 5c)			
V-4	Frontal bone	CH	Cut mark	Right squama	Long lesion (34 × 4 mm), with flaking on the antero-medial margin and several microstriae on the bottom; the lateral margin is particularly sloping and seems altered by subsequent scraping actions; the posterior end is shallower and thinner (1 mm) than the anterior one (Fig. 6a, b, c)	May the lesions be related to powerful defleshing or violent actions? Interpersonal or ritual violence?		
			Chop mark	Right squama	Chop mark (18 × 3 mm), more irregular and deeper; the anterior part of the groove is shallower and slightly laterally curved; the posterior end suggests a cutting action revealed by microstriae on the bottom; sediments fill the bottom of the lesion demonstrating its antiquity (Fig. 6b, c, d)			
			Thin cut marks	Right squama	At least four thin cut marks (maximum length 8 mm), located posteriorly to the chop mark (Fig. 6d)	Defleshing of the cranium		
C1-6	Calotte	M, AD	Cut marks	Left frontal squama	Cut mark (7 mm) with microstriae on the bottom Posteriorly to the cut mark, another small lesion (2 mm) is visible	Defleshing of the cranium by severing the epicranius	Incomplete fracture with adhering flake (20 × 12 mm) on the left frontal bone and incomplete and depressed fracture (40 mm) on the occipital bone in correspondence to postmortem breakages Circular incomplete and depressed fracture (35 × 28 mm) on the left parietal bone, interrupted anteriorly by a lacuna of irregular shape (35 × 23 mm), with depressed areas on the margins and postmortem damages	Fractures on frontal and occipital bones could be accidental and/or due to post depositional damage Patterned injury, likely due to blunt force trauma on the parietal bone, indicating possible violent actions (interpersonal or ritual violence?)
C1-7	Calotte	F, AD	Scrape mark	Right parietal	Scraped area in correspondence to the removal of a bone flake (8 × 3 mm), with several striae on the bottom (Fig. 7a)	Powerful defleshing, causing the detachment of a bone flake	Incomplete fracture of the frontal bone just above the right fronto-zygomatic suture, with a small radiating fracture. Two incomplete fractures perpendicular to the right orbital margin converging at about 10 mm above the orbit A large lacuna of irregular shape (78 × 60 mm) involves the left parietal and smaller portions of the right parietal and occipital bones (Fig. 7d); in its superior part, a triangular flake of bone is still attached (Fig. 7e); some incomplete radiating fractures. The border of the lesion as well as its endocranial surface display postmortem damages	The fractures present the features of blunt force trauma, indicating possible violent actions (interpersonal or ritual violence?)
			Cut mark	Occipital	Cut mark (7 mm), transversally oriented on the left supreme nuchal line, attachment of the occipital belly of the occipitofrontalis muscle; the lateral margin is straight, while the medial one is more irregular and oblique with flaking (Fig. 7b)	Defleshing by severing the occipitofrontalis muscle		
			Chop mark	Right mastoid process C2-SN*	Chop mark (14 × 3 mm, depth 3 mm), transversally oriented (Fig. 7c), in correspondence to the sternocleidomastoid muscle enthesis	The cutting of the sternocleidomastoid muscle allows the detachment of the skull from the trunk In a fleshed corpse, a chop reaching the mastoid could also be related to the attempt to detach the mandible		
V-AMH-200	Mandible	M, YA	Cut mark	Left condylar neck	Cut mark (3 mm) on the mandibular notch, in correspondence to the temporomandibular joint capsule	Disarticulation of the mandible	Incomplete fracture on the inner surface of the left ramus departing from under the coronoid process (which is broken and glued) and descending inferiorly to the mylohyoid line The left condyle is preserved	Considering the cut mark on the mandibular notch, the incomplete fracture may have been produced during disarticulation of the mandible
V-3B	Mandible	M, AD	-	-			A semi-circular incomplete fracture line is visible on the right retromolar fossa. Another incomplete fracture on the inner surface of the right ramus departs from the previous one, crosses the mylohyoid line and reaches the inferior border The right condyle is not preserved, but the fracture surface is covered by consolidant	Incomplete fractures, possibly due to disarticulation of the mandible
C2-13	Mandible	CH	-	-			Fracture with possible peeling in the left part of the corpus of the mandible, exposing the crown of M2 still included in the alveolus	Fresh bone breakage, possibly due to dismembering practices

Abbreviations: *M* male, *F* female, *AD* adult, *YA* young adult, *CH* child

^*^The right mastoid process C2-SN was stored in a different box but articulates with calotte C1-7

In the postcranial skeleton, perimortem lesions have been observed in all skeletal districts (three vertebrae, four ribs, one scapula, four humeri, one ulna, one ischium, three patellae, three tibiae, two fibulae, one calcaneus, one talus), but especially on femurs (14 specimens). Most of them are linked to sharp force trauma, interpretable as cut (Fig. 8) and chop marks, crushing and perforating/penetrating lesions (Fig. 9). Moreover, some long bones (one adult humerus, 11 femurs, one adult and one adolescent tibia along with one adult fibula) show some typical features of possible fresh bone fractures, but often in association with dry or mineralised bone breakage characteristics (Fig. 10). A detailed description of each specimen is provided in supplementary Table S2.

## Discussion

### Radiometric dates

Results of the radiometric analyses (Table 1) allow to attribute the studied sample to a final phase of the Neolithic and an early phase of the Eneolithic period in northern Italy (Miari et al. 2022), as it was already supported by L. Fantini.

In Italy, the final phase of the Neolithic is identified in a large part of the central area of the Po Valley with the *facies* of S. Ilario (Reggio Emilia), recognised by Lawrence Barfield in the 1970s (Barfield 1975). This *facies* is currently identifiable in eastern Lombardy, eastern lower Veneto, western Romagna and Emilia (Ferrari et al. 2017). In Emilia, the dates available for this period refer to the first quarter of the IV millennium BC (Bernabò Brea et al. 2017). The transition from the Final Neolithic to the Eneolithic in northern Italy is subsequently dated to 3600 cal BC, while the Early Eneolithic between 3600 and 3300 cal BC (Dolfini 2010). This chronology is also attested in some of the earliest Eneolithic sites in Emilia Romagna (Steffè et al. 2017), as well as in the surrounding areas of the ‘Gessi della Croara’ (Nenzioni and Lenzi 2022).

### State of preservation, MNE, MNI, ERI and biological profile of the individuals

The mean percentage of collagen content in prehistoric and historic bones from the assemblage is similar (9.9% and 10.9% respectively). These values are lower than those reported in literature for the bone tissue (22–23% by dry weight is represented by organic matter, 90% of which consisting of collagen; Turner-Walker 2008), demonstrating that a certain amount of the organic component has been lost. Given the recent chronology of the historic specimens (seventeenth–twentieth century AD; Table 1), we can infer that in this karstic environment the diagenetic process is rather fast. This implies that both dry and mineralised fractures could have been produced in ancient times, for accidental reasons or following human interventions some time after death.

Due to the high fragmentation of the materials, as well as the presence of bones still included in sediments and thus not counted, the MNE, MNI and ERI (Table 3) are in all likelihood underestimated, being it difficult to confirm all the possible associations among the fragments of each skeletal district.

The overall ERI of most of the bones may appear rather low. However, all the skeletal districts are attested and, even if adult left elements are slightly more frequent than right ones (Table 3), this difference seems too small to support an intentional selection of left elements. The most robust elements, such as humeri and femurs, are the better-attested, while the most fragile elements, such as sternum and vertebrae, are underrepresented. The smallest hand and foot bones are clearly underrepresented but still present in a coherent number. The loss of so many elements can be attributable to different causes, such as inaccuracies during the recovery, that surely occurred for the Farneto rock shelter, as well as the impossibility to count and study many fragments still embedded in sediment blocks (Fig. 2c, d). As gnawing traces were found, it is possible that carnivore activity may have also contributed to the loss of bone elements. Moreover, the most fragile bones (e.g. sternum, vertebrae) easily undergo natural destruction because of their internal structure and composition, also taking into account the unstable karstic environment of the area. The latter could account for the high degree of fragmentation of bones, as shown by the large number of fragments that measure less than 50 mm (Fig. 3).

The smallest bones (e.g. hand and foot bones) could have been lost also following intentional displacements of the remains for ritual or practical reasons (Knüsel et al. 2016). These displacements could have been the result of secondary depositions from different primary deposits or of rearrangements of bones within the original place of disposal. Both these possibilities are attested in other roughly coeval sites in Emilia Romagna (Miari 2013; Cavazzuti 2018). The presence of bones from all skeletal districts is consistent with the hypothesis that at least some of the bodies were placed fleshed in the original place of deposition and then underwent manipulation practices.

The comparison of the ERI from the Farneto rock shelter with other archaeological contexts for which the type of deposition is known and the excavation documentation is clear, allows for some considerations about the possible interpretation of the Farneto assemblage as a funerary context. The sites used for comparison are Scaloria Cave (Foggia, Apulia, southern Italy, Neolithic; corpse manipulation and secondary depositions), Kunji Cave (Iran, Bronze Age; collective and secondary burials with probable bone selection) and West Tenter Street (London, UK, Roman period; single primary inhumations; data from Knüsel et al. 2016; S3). Interestingly, the Farneto rock shelter, Scaloria Cave and Kunji Cave display the same ERI distribution pattern, while West Tenter Street presents a better representation of the vertebrae, sternum, hand and foot bones, as expected for primary inhumations. Despite some minor differences, that could be influenced by the different recording method or stochastic sampling of the material due to taphonomic processes and excavation methods (S3), this shared pattern supports the hypothesis that secondary burials were present at the Farneto rock shelter.

Regarding sex and age at death, both male and female individuals and all age classes are attested, so that a horizontal intentional selection of the individuals to be buried can be excluded. The paucity of perinatal or very young infant remains, testified by one single specimen (the complete left humerus C4-61), can be explained by several reasons that are not mutually exclusive: their natural fragility (Gordon and Buikstra 1981; Guy et al. 1997), their misidentification during recovery (Schaefer et al. 2009), possible underlying ideologies and/or funerary practices that originally led to their exclusion (Guy et al. 1997; Cveček and Schwall 2022).

### Bone colour, burning and gnawing traces

#### Colour

During the study of the assemblage, in the lack of any information regarding the original position of the human remains, bone colour and staining could have contributed to the reconstruction of the original disposition of the skeletal materials. The vast majority of the bones have a very light white colour, compatible with a permanence in an alkaline soil, while only a few elements present a brown colour, characteristic of an acidic soil (Turner-Walker 2008). In fact, radiometric dates have demonstrated that the latter are extraneous to the prehistoric context, being much more recent (Table 1), suggesting the hypothesis of microenvironmental changes through time in the site influencing the colour but not the rate of collagen degradation. The interpretation of the light reddish specimens is difficult, but we can propose that subadult bones react differently to the microenvironment of deposition, perhaps with a different chemical exchange with soil substances (Guy et al. 1997). Anyway, this colour is more similar to the light colour of the majority of other bones than to the brown colour of the more recent specimens excluded from the study.

#### Burning

The circumscribed reddish brown stains are compatible with an estimated fire temperature between 285 and 525 °C (stage 2 according to Shipman et al. 1984; Fig. 4a). Besides temperature, other factors could affect fire-induced bone modifications, such as environment (oxidising or reducing conditions), time of exposure, dry or fresh status of the bone. In our case, the absence of modifications other than colour suggests that bones were already dry when affected by fire (cf. de Becqdelievre et al. 2015). The small dimensions of stains along with the low frequency of burned remains support the hypothesis of an accidental contact with fire. In fact, in the Eneolithic Italian sites (in Tuscany, Grifoni Cremonesi 2001; Lombardy, Barfield 2007; Emilia Romagna, Cavazzuti 2018) where cremation is considered part of the funerary ritual, the percentage of burned skeletal remains is much higher.

#### Gnawing

The total number of bone elements affected by gnawing activity may have been underestimated due to the state of preservation of some skeletal remains (e.g. surface covered by incrustations). Nevertheless, some rodent (Fig. 4b) and carnivore (Fig. 4c, d, e) gnawing traces were found. In the case of the adolescent right tibia (C7-AMH-223; Fig. 4d), the two punctures are compatible with the dentition (distance between maxillary and mandibular canines) of *Canis familiaris*, *Canis lupus* or *Ursus arctos* (Murmann et al. 2006; Pokines 2013), animals that inhabited the Farneto area in prehistoric times. In the surrounding areas, such as in the nearby territory of Monterenzio Vecchio (Bologna), the presence of the three species is documented from the Eneolithic to the Bronze Age (Sala 1980; Maini 2012; Ciucani et al. 2019). The most ancient specimen of *Canis lupus* was found in the Neolithic site of Razza di Campegine (Reggio Emilia; Cazzella et al. 1976), while several canid remains were found in Bronze Age sites all over the region (Farello 1995; De Grossi Mazzorin 1996; Koupadi et al. 2020), occasionally accompanied by *Ursus arctos* remains, such as in the sites of Crocetta di Sant’Agata Bolognese (Bologna; Maini 2012) and Monte Castellaccio (Bologna; De Grossi Mazzorin 1996). Moreover, skeletal remains of *Ursus arctos* were recognised among the human remains from the Farneto rock shelter in the 1990s by Gianni Giusberti (Facchini et al. 1999). All these results are coherent with an original deposition inside a cave environment, where human bones were likely exposed or buried in shallow pits, easily reached by wild animals (Pokines 2013).

Within the overall sample, several irregular-shaped depressions that mimic carnivore pits may be detected, in particular on cranial vault surfaces (e.g. frontal bone C1-9), but they are more likely the result of marks left by falling blocks or produced during the rolling of the bones, which probably originated from a higher place (Fernández-Jalvo and Andrews 2011).

### Perimortem lesions

The presence of several perimortem lesions on both cranial and postcranial remains suggests that the Farneto prehistoric group performed practices of treatment of the cadaver on at least some individuals of both sexes and all age classes. These practices encompassed various activities such as disarticulation (lesions related to joint structures, i.e. epiphyses, entheses of ligaments, capsules or tendons stabilizing some joints) or dismemberment (bone fractures, deep chop marks on epiphyseal regions or on diaphysis) and scarnification/defleshing (cut marks or chop marks in correspondence to large muscular masses, as well as fine cuts aimed at cleaning the bones even from thin residues of soft tissues or just the periosteum; Table 4; S2). In some cases, episodes of interpersonal violence or of violent actions on cadavers (e.g. ritual sacrifices, anthropophagic practices) can be hypothesised.

**Fig. 5:**
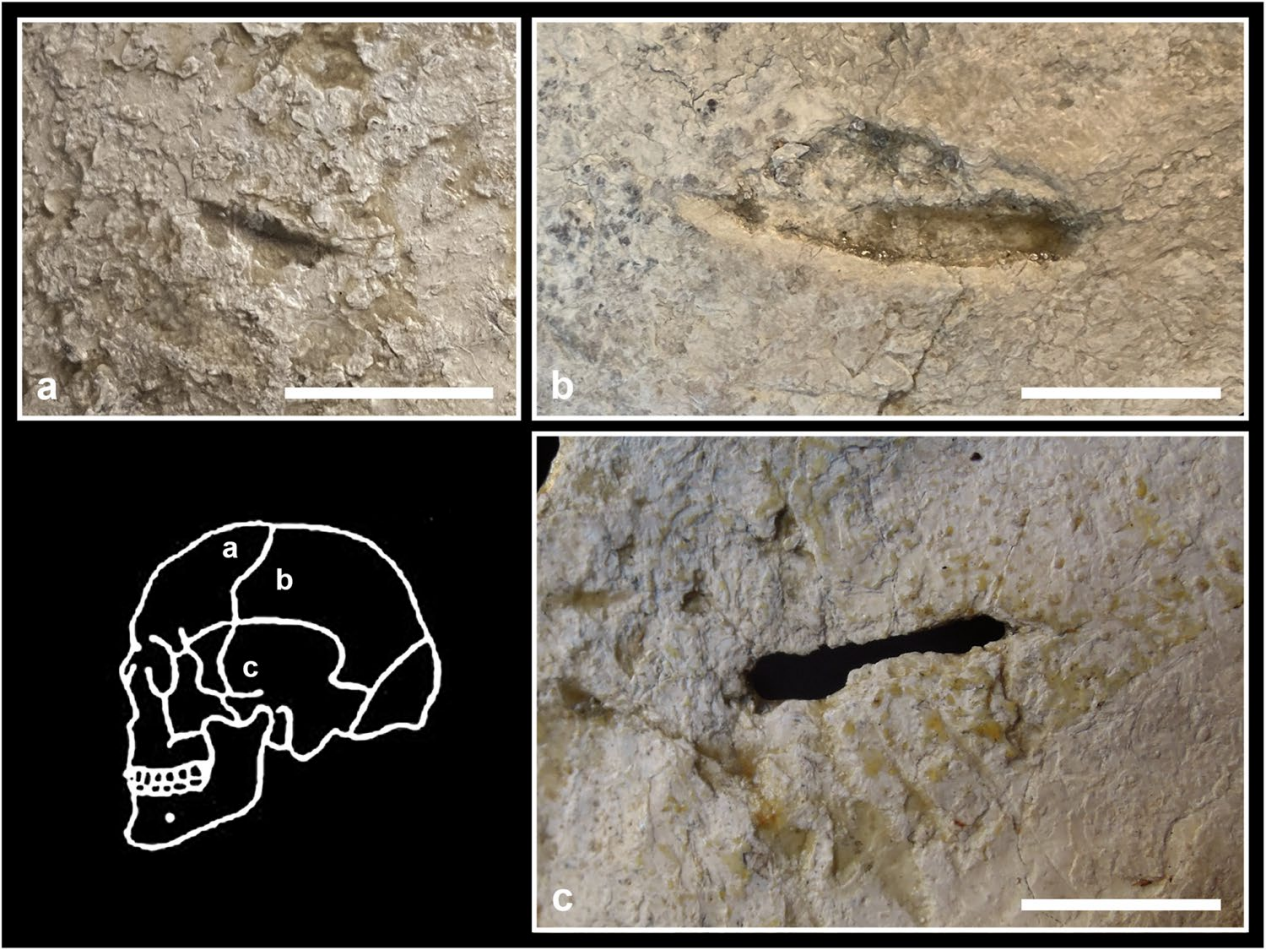
Perimortem lesions on the adult calotte V-3: **a** oblique chop mark on the frontal bone covered by resin; **b** chop mark on the parietal bone covered by resin with the removal of a bone flake superiorly; **c** perforating lesion on the left temporal bone crossed by three thin cut marks. Bar = 10 mm

Due to the fragmentation of the bones and the state of preservation of their surfaces and fracture margins (e.g. incrustations, decortication, but also the use of resins and consolidants during previous studies; Fig. 5), many lesions could not have been preserved or their characteristics are no longer discernible and interpretable. The fact that perimortem lesions were found especially on femurs could be also the result of sampling bias given the robusticity of this bone. For these reasons, we did not calculate the frequency of lesions and the most affected skeletal districts. It is thus possible that their presence has been underestimated.

The morphology of many cut marks (e.g. flaking of borders, shoulder effect, parallel microstriae on the bottom of the sulcus; Fig. 8) is typical of stone tools, more or less retouched (Greenfield 1999; Moretti et al. 2015). Indeed, copper blades have not been found in the site.

As regards crania, our results allow hypothesising disarticulation/dismembering of both the mandible from the cranium and the cranium from the trunk, as well as defleshing (Table 4). Lesions such as the chop mark on the right mastoid process (C1-7; Fig. 7c), as well as the lesions detected on mandibles, may be the result of the attempt to disarticulate the mandible from the cranium (Table 4). In the first case, the lesion is also compatible with disarticulation of the cranium from the column, as are the lesions found on two cervical vertebrae (Fig. 8a, b; S2).

**Fig. 6:**
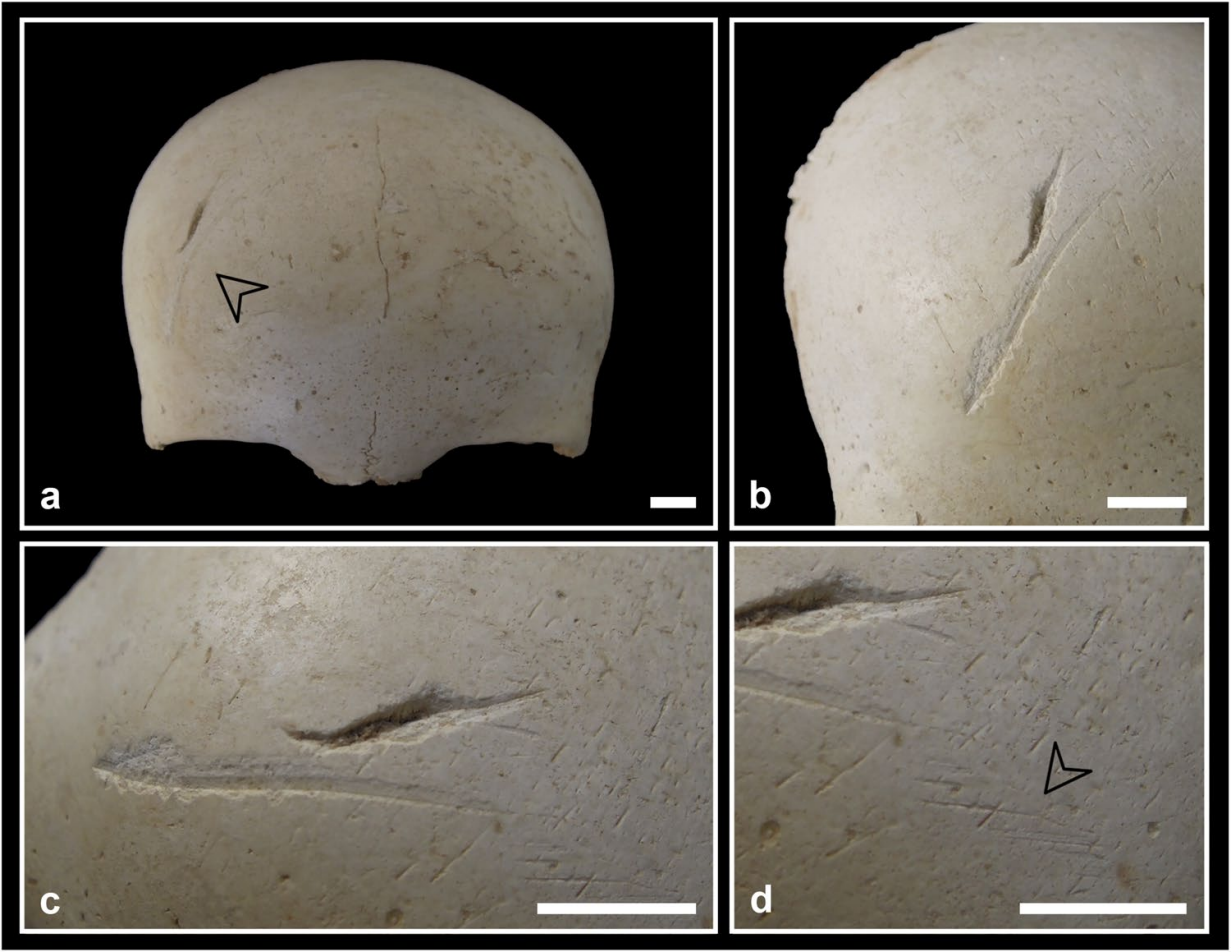
Perimortem lesions on the child frontal bone V-4: **a** position of the lesions indicated by the arrow; **b** overall view of the lesions; **c** detail of the biggest lesions, consisting of one long cut mark (note flaking, microstriae and scraping traces) and one chop mark (note microstriae and sediments filling the lesion); **d** detail of the thin cut marks posteriorly to the chop mark. Bar = 10 mm

The presence of cut marks of various dimensions (from very thin, barely perceptible lesions, to long and deep ones, e.g. on frontal bone V-4; Fig. 6) on the cranial vaults suggests the presence of cleaning practices performed with different degrees of strength. Some lesions (especially, but not only, patterned fractures; Kimmerle and Baraybar 2008; Table 4) seem to indicate violent actions whose aim cannot be ascertained (interpersonal vs. ritual forms of violence, on the living person vs. on the cadaver), even if accidental trauma (e.g. falls, falling rocks) cannot be excluded (fractures in V-2B, frontal and occipital fractures in C1-6).

**Fig. 7:**
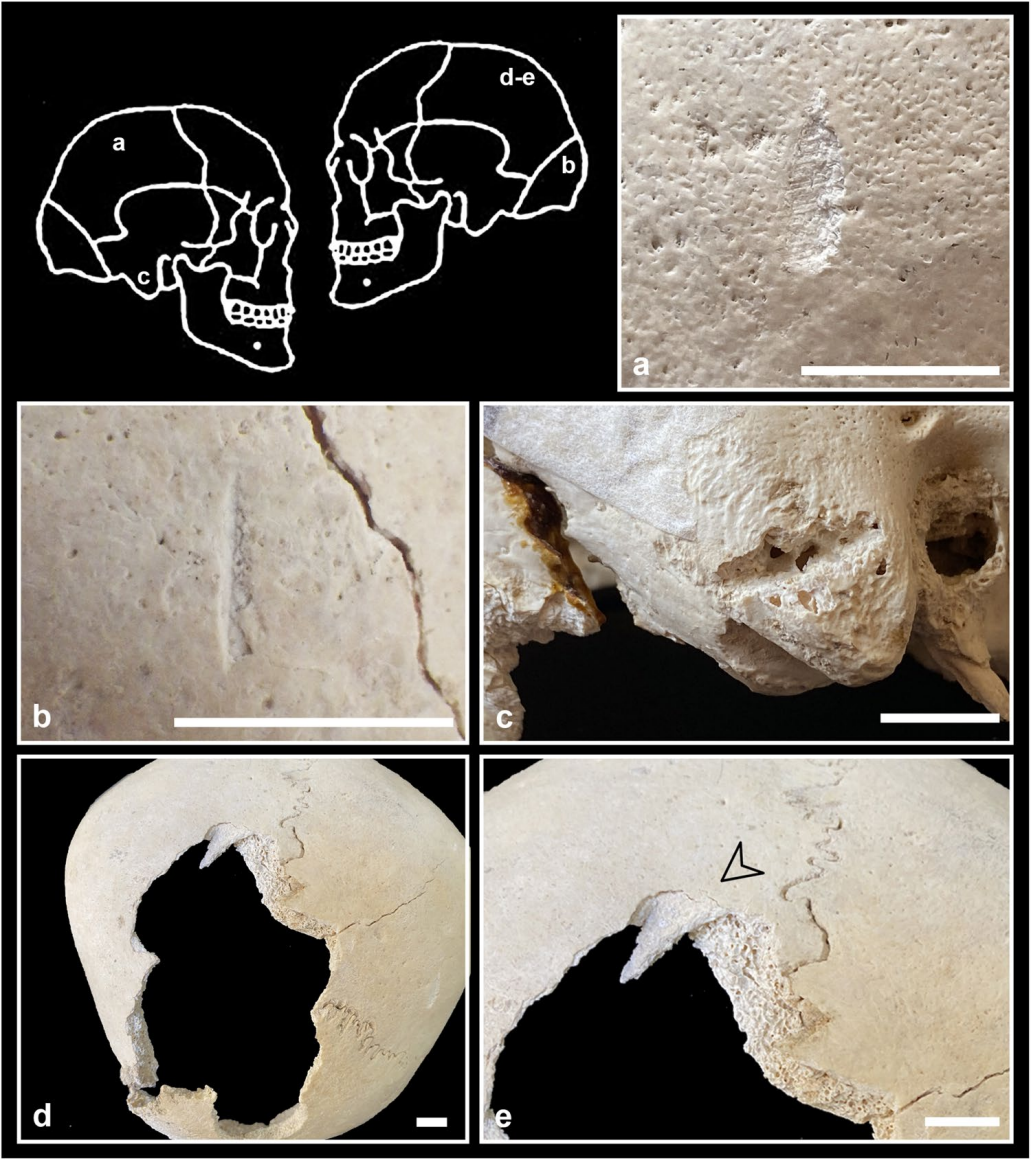
Perimortem lesions on the adult calotte C1-7: **a** scrape marks and removal of a bone flake on the right parietal bone; **b** cut mark transversally oriented on the supreme nuchal line (note the straight lateral margin and the medial one with flaking); **c** chop mark transversally oriented on the right mastoid process;** d** large lacuna of irregular shape involving most part of the left parietal bone and some portions of the right parietal and occipital bones (note the incomplete radiating fracture on the right parietal bone); **e** detail of the triangular bone flake still attached at the cranium on the superior portion of the lacuna. Bar = 10 mm

In two cases, the particular features associated with the lesions (the cut marks in correspondence to the perforating lesion in V-3; Fig. 5c; and the morphology of the margins of the lacuna in V-2; Table 4) could suggest some form of therapeutic interventions on previous wounds. The absence of traces of bone reaction attests that the individuals did not survive. Medical surgery has been hypothesised for the coeval cranium of the Marcel Loubens Cave, located not far from the Farneto rock shelter (Belcastro et al. 2021), and for other Italian crania dating back to Neolithic times (Germanà and Fornaciari 1992; Formicola et al. 2012).

**Fig. 8:**
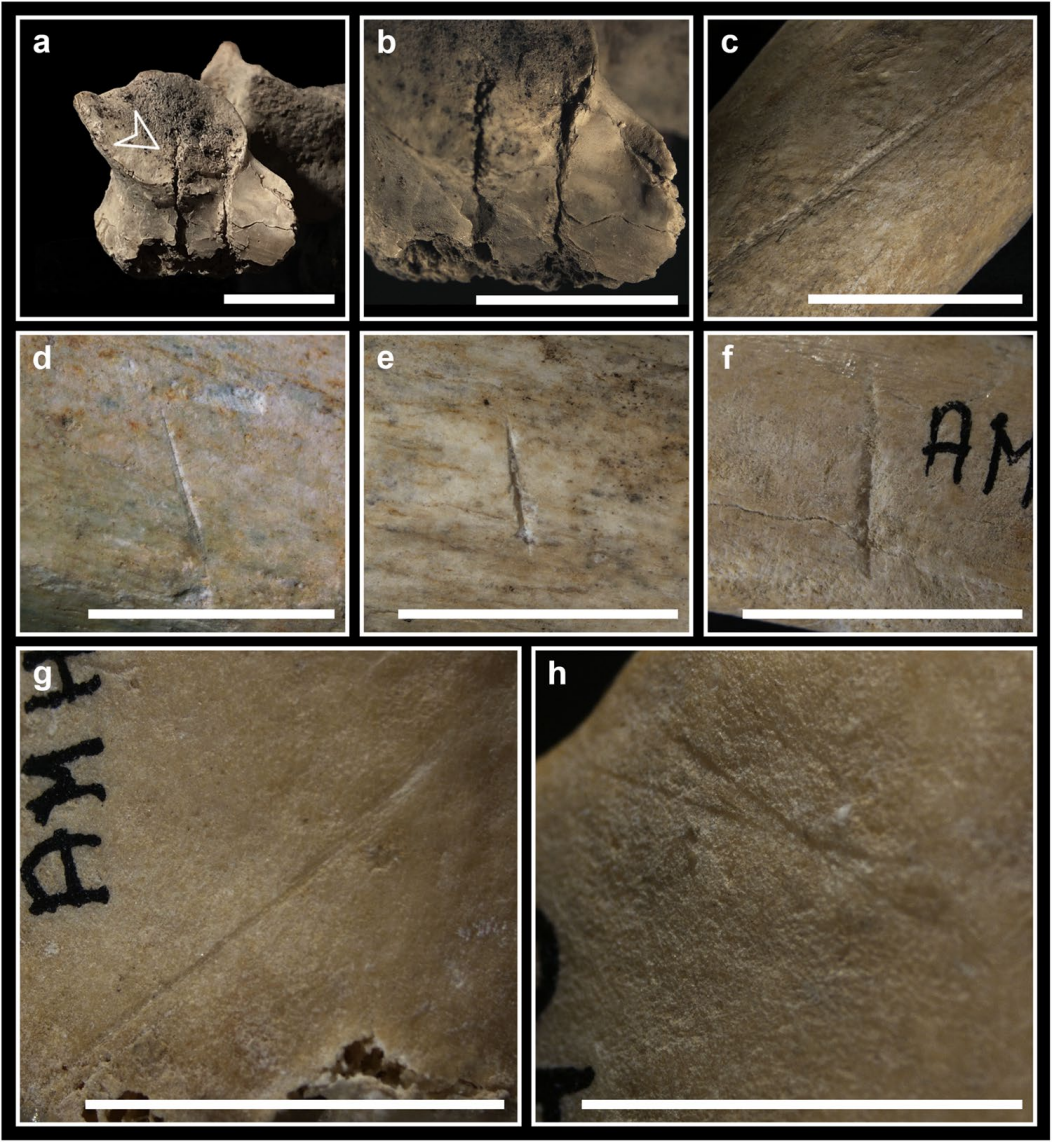
Perimortem cut marks on postcranial bones (detailed description in S2): **a** two cut marks on the left superior articular surface of the cervical vertebra C5-BL3-SN; **b** detail of the same two cut marks on the cervical vertebra C5-BL3-SN (ph. stereomicroscope); **c** cut mark on the inner surface of the body of the rib C1-AMH-270 with microstriae on the bottom (ph. stereomicroscope); **d** cut mark filled with sediments at least in one portion on the diaphysis of the humerus C7-AMH-232 (postero-lateral view; ph. stereomicroscope); **e** cut mark on the diaphysis of the same humerus C7-AMH-232 (postero-lateral view; ph. stereomicroscope); **f** cut mark on the diaphysis of the humerus C7-AMH-236 (posterior view; ph. stereomicroscope); **g** cut mark on the medial articular surface of the patella C7-AMH-246 with microstriae on the bottom (ph. stereomicroscope); **h** cut marks forming a X-shape on the medial articular surface of the same patella C7-AMH-246 (ph. stereomicroscope). Bar = 10 mm

The lesions detected on the postcranial bones suggest that corpses were disarticulated and/or dismembered and cleaned from soft tissues (S2). Lesions in correspondence to the epiphyses are likely related to disarticulation. Regarding the timing of treatment, it is useful to distinguish between persistent and labile joints, because lesions on the latter imply an intervention immediately or a short time (depending on environmental conditions) after death (Duday 2009; Mickleburgh and Wescott 2018). In our case, labile joints (e.g. hands, toes, cervical vertebrae, patellae) are not much represented, but some lesions have been detected on a cervical vertebra, a femoral head and on the patellae. Thus, it is possible that at least some corpses were treated soon after death.

**Fig. 9:**
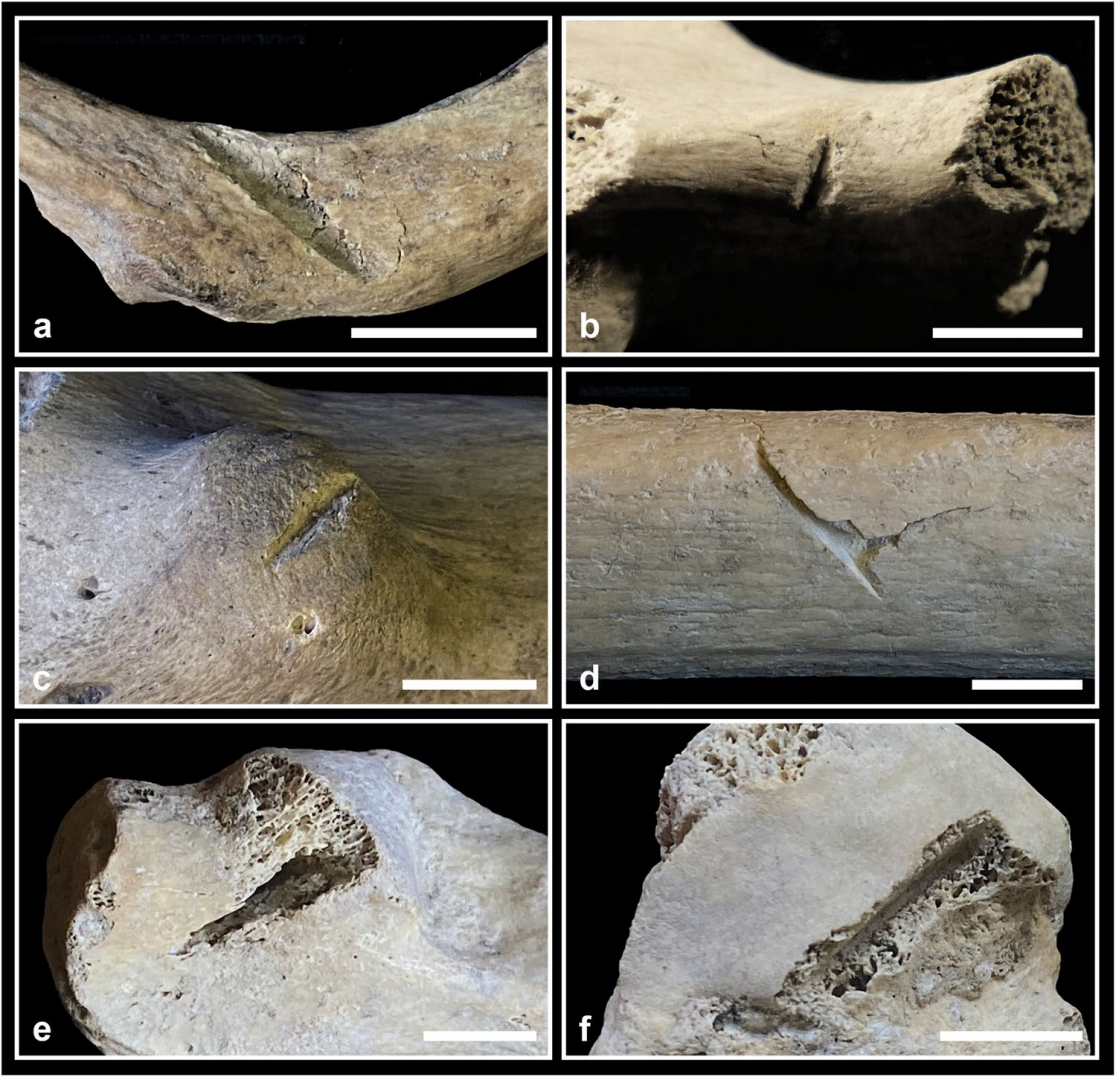
Perimortem sharp force lesions on postcranial bones (detailed description in S2): **a** crushing lesion on the superior surface of the rib C1-AMH-269 anteriorly to the tubercle; **b** chop mark on the anterior portion of the ramus of the ischium C8-114; **c** chop mark filled with sediments on the lesser trochanter of the femur V-AMH-211; **d** chop mark on the diaphysis of the femur C5-BL2-SN with a partially detached large flake of bone still adhering; **e** penetrating lesion on the calcaneus C4-159; **f** penetrating lesion laterally to the calcaneal anterior articular surface of the talus C4-166. Bar = 10 mm

The lesions seem to have been produced by different tools and in different manners. In particular, where sharp force lesions are associated with crushing (some vertebrae, ribs; Fig. 9a; scapula and some long bone epiphyses), it is possible that a blunted blade acted where cancellous bone is covered by a thin layer of cortical bone. Another possibility is that the presence of soft tissues protected in some way the bone from the blade, causing crushing instead of clear cuts during corpse treatment (cf. Shipman and Rose 1983). As regards thin cut marks, these suggest the use of sharp blades during cleaning practices (Fig. 8g, h). The chop marks, instead, indicate powerful actions during cleaning of fresh cadavers with thick muscular masses or during dismembering practices (Fig. 9b, c, d), that in some cases could have caused bone breakage (e.g. humerus C4-58; S2).

**Fig. 10:**
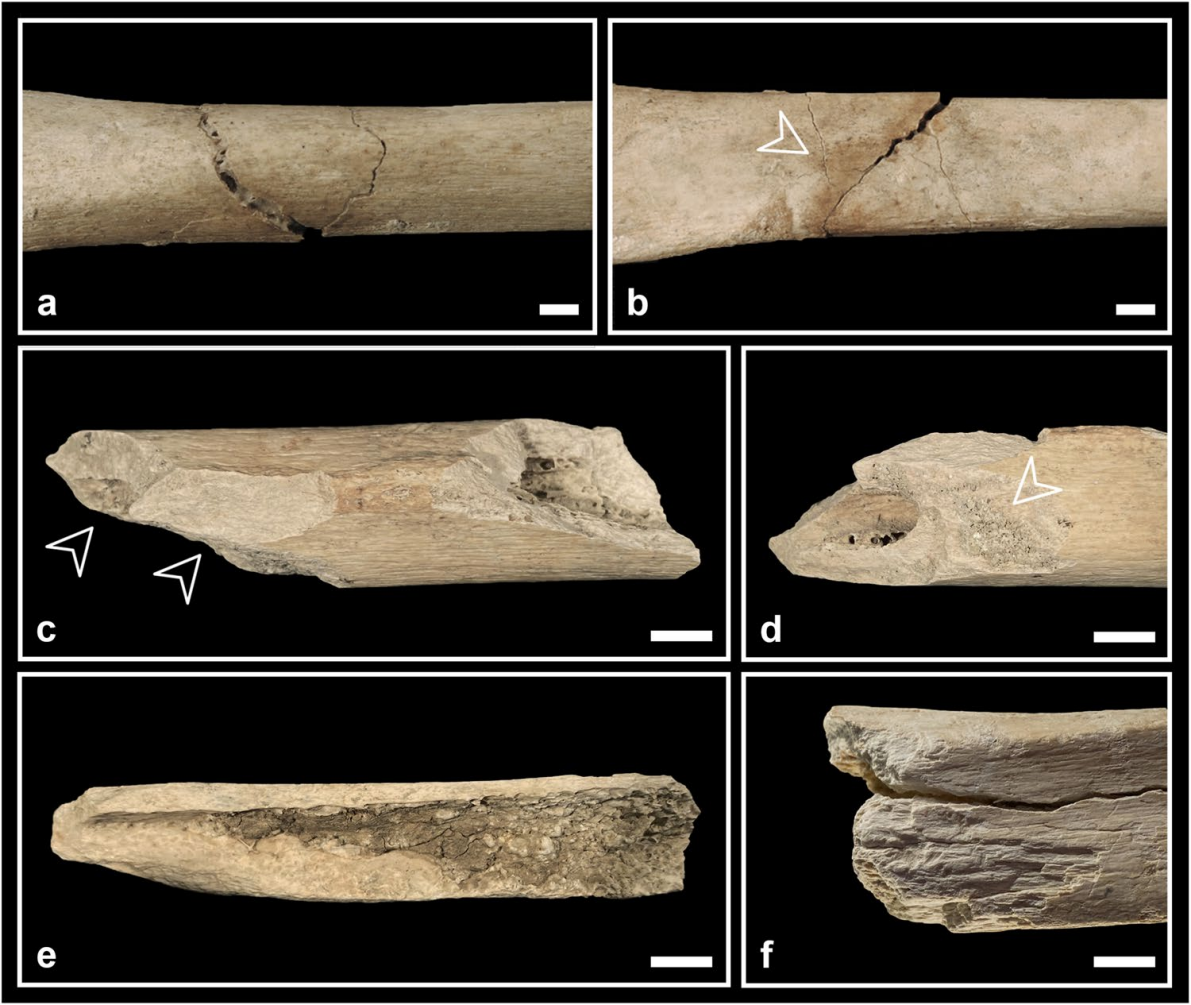
Perimortem fractures on postcranial bones (detailed description in S2): **a** butterfly fracture on the tibia C7-AMH-223 (anterior view); **b** the same butterfly fracture on the tibia C7-AMH-223 from which an incomplete fracture line departs (posterior view);** c** femur C5-BL1-SN showing spiral fractures with spalling of cortical bone; **d** the same femur C5-BL1-SN presenting some sediments adhering at one of the spalling areas; **e** bone flake C5-AMH-79 showing an oval profile, smooth fracture surfaces and acute proximal fracture angle, probably resulting from a fresh bone breakage; **f** peeling of the tibia C4-AMH-298 with roughened exfoliated surface. Bar = 10 mm

A few elements present typical fresh bone fractures (Fig. 10; S2). In one case (the complete right tibia C7-AMH-223; Fig. 10a, b; S2), a butterfly fracture has been observed, which is considered a typical perimortem fracture (Cappella et al. 2014; Reber and Simmons 2015). In addition, the femoral diaphysis fragment C5-BL1-SN (Fig. 10c, d) and the bone flake C5-AMH-79 (Fig. 10e) present a pattern of features (spiral outline, smooth fracture surface and acute fracture angle) considered indicative of fresh bone breakage. Moreover, the presence of peeling (Fig. 10f) or spalling (Fig. 10c, d) of the cortical surface in correspondence to bone breakages is reported in the literature as indicative of perimortem fractures (Andrews and Fernández-Jalvo 2012; Pickering et al. 2013; Knüsel and Robb 2016); therefore, it possibly occurred during corpse treatment. In the frame of mortuary corpse treatment, intentional fresh bone breakages are more likely related to dismembering practices. Thus, the contextual presence of sharp force lesions interpreted as such reinforces the hypothesis that interventions on cadavers were at least partially responsible for those bone fractures as well. Nevertheless, other taphonomic factors may also produce similar traces, as it has been noticed as a result of *Ursus arctos* gnawing and scavenging activity, leaving on the bones fracturing, peeling, crenulation, tooth pitting and scoring (Arilla et al. 2014).

However, some other fractures present typical fresh bone features together with dry or mineralised bone breakage characteristics, making their interpretation doubtful (S2). Given the fast diagenetic process probably characterising the context, so that bones rapidly became dry or mineralised, and the presence of lesions on persistent joints, these fractures may have been produced during other secondary manipulation practices performed some time after death. Considering the instability of the karstic environment of the site, postmortem accidental events could account for further damage as well.

### The Farneto rock shelter assemblage in the Italian and European framework

The paucity of faunal remains and the analysis of the ERI suggest that the Farneto rock shelter human bones may be referred to a funerary assemblage, probably with the presence of secondary practices. Eneolithic funerary contexts in Emilia Romagna are represented by both necropoles/isolated pit graves and burials inside natural cavities (Cardarelli 1992; Miari 2013, 2018; Cocchi Genick 2014). Thanks to the results of the radiometric analyses, the obtained absolute dates from the Farneto rock shelter demonstrate an early use of natural cavities for funerary purposes in the central area of the region. This early frequentation is also confirmed by other absolute dates recently obtained at the MPLD in San Lazzaro di Savena, from materials from the surrounding areas of the ‘Gessi della Croara’ (Nenzioni and Lenzi 2022). These are two human specimens from ‘Cava I.E.C.M.E.’ and ‘Grotta dell’Ossobuco’, dating to 3800–3642 cal BC and 3475–3370 cal BC respectively. For this period, the available comparisons in Italy are few. An example is represented by the site of ‘Poggio di Spaccasasso’ (Grosseto, Tuscany, central Italy), which had a funerary use from the second quarter of the IV millennium BC throughout the entire Eneolithic period (Volante 2018; Volante and Pizziolo 2019). Another comparison may be found in the site of ‘Grotta Bella’ (Terni, Umbria, central Italy), where the use of the cave for funerary purposes during the Neolithic was recently hypothesised in the so-called ‘Sala dello Scheletro’ (Larocca 2022).

Due to the circumstances of the discovery and the retrieval of the human skeletal remains from the Farneto rock shelter, it is not possible to document which type of burial or deposition (primary, secondary, collective or a mixture of all of them) the individuals originally had. Most skeletal remains are fragmented and they appear commingled and chaotically disposed also in the sediment blocks that keep together some fragments still today (Fig. 2c, d). The archaeological objects cannot be interpreted as grave goods because of their uncertain origin and collocation. In any case, given the chronology of the skeletal assemblage to the Late Neolithic/Early Eneolithic period, grave goods were more probably represented by the ornamental elements found in the site (Fig. 2a), rather than the pottery objects which likely date to subsequent periods of frequentation (Nenzioni and Lenzi 2022).

Given the conformation and nature of the deposit (Fig. 1a, b), it is possible, as L. Fantini originally thought, that the materials originated from a higher place, a natural cavity likely used for funerary purposes, and slipped down during ancient times and in more recent years due to mudslides and rockfalls. Due to these events, which are typical of the karstic environment characterising the ‘Parco dei Gessi Bolognesi e Calanchi dell’Abbadessa’, the front of the Farneto hill facing the Zena stream has retreated some tens of metres compared to prehistoric times (Nenzioni and Lenzi 2022).

The analysis of perimortem lesions suggests that the individuals from the Farneto rock shelter were subjected to disarticulation practices and intentional cleaning from soft tissues. Although the original type (or types) of deposition cannot be ascertained, corpse manipulation, as well as the ERI distribution pattern, is consistent with the practice of secondary burial. This evidence can be related to our knowledge about Eneolithic funerary practices in central and northern Italy (Cocchi Genick 2009, 2014) and in Emilia Romagna in particular (Miari 2013; Belcastro et al. 2021; Miari et al. 2022). However, the presence of at least some primary burials cannot be excluded. To summarise, different scenarios could be hypothesised, not mutually exclusive:

1. Original presence of some complete or partial primary burials that underwent destruction due to natural events;
2. In situ body manipulation and subsequent secondary disposal of some of the bones in other areas of the site or elsewhere;
3. Secondary deposition of bones from corpses treated elsewhere and transported into the site.

With regard to the funerary rituals performed in the Eneolithic contexts in Emilia Romagna, both inside natural cavities and in pit graves necropoles, the presence of dislocated, commingled or even isolated human remains is attested and is commonly interpreted as the result of intentional manipulation practices (Miari 2013, 2018; Cocchi Genick 2014; Cavazzuti 2018; Cavazzuti et al. 2020).

As concerns natural cavities, the use of these contexts as secondary burial places is already well known. In the nineteenth century, Don Gaetano Chierici first discovered human remains in the ‘Tana della Mussina’ (Reggio Emilia), where most skeletons appeared disarticulated and some of them were partially burnt. The context was interpreted as a collective burial that was frequented at various times (Tirabassi 2013). The comparison between the ERI distribution of the Farneto rock shelter and the one from the ‘Tana della Mussina’ (data from Cavazzuti et al. 2020) highlights the typical features of secondary deposits in both sites, but a different representation of some skeletal districts (S4). In particular, in the ‘Tana della Mussina’ crania are less represented than mandibles, while in the Farneto assemblage they are equally present. Moreover, in the ‘Tana della Mussina’, the percentage of lower limb bones is particularly low (less than 35%), while that of upper limb is particularly high (more than 70%). Thus, in this site, the high representation of mandibles and upper limb bones could suggest an intentional selection of districts to be stored in different places or treated in different manners. In the Re Tiberio Cave (Ravenna), collective burials were present as well, hosting disarticulated and displaced bones. Here, the almost complete absence of crania is noteworthy, indicating an intentional selection of bones (Miari 2013; Cavazzuti 2018). Finally, in the ‘Tanaccia di Brisighella’ (Ravenna), two subadult crania were placed in a niche together with a vase (Miari 2013). Moving to pit graves necropoles, manipulation of the crania along with the upper part of the skeleton is attested in the necropolis of ‘Celletta dei Passeri’ (Forlì-Cesena; Miari et al. 2017). Secondary depositions are also attested in the context of ‘Fornace Cappuccini’ (Ravenna; Antoniazzi et al. 1990).

Such funerary practices, involving dislocation and commingling of the human remains, have been interpreted as part of a cult of the ancestors, where the individual is depersonalised through fragmentation and mixing with others, aimed at reinforcing social cohesion and collective memory (for central and northern Italy cf. Conti et al. 2006; Cocchi Genick 2009, 2014; Miari 2013, 2018).

Besides any possible ritual interpretation, the available literature regarding these funerary contexts mostly describes practices of selection, displacement and commingling of human remains as postmortem interventions performed on skeletonised remains. On the contrary, as regards the perimortem treatment of corpses, there is not much evidence from Neo/Eneolithic Italian funerary contexts. This may be due to the absence of available documentation and anthropological examination for the sites excavated during the last centuries. In fact, most recent analyses are radically changing this scenario. The taphonomic and anthropological analyses carried out on the isolated Early Eneolithic cranium recently retrieved in the Marcel Loubens Cave allowed the identification of some peculiar funerary practices, such as cleaning from soft tissues immediately after death, i.e. perimortem (Belcastro et al. 2021). Similar results come from the recent restudy of the human skeletal remains from the ‘Tana della Mussina’, where it was possible to detect the presence of perimortem cut marks on a mandible (Cavazzuti et al. 2020), and possibly from ‘Fornace Cappuccini’ (still under study; cf. Belcastro et al. 2021). In this framework, the results of the anthropological restudy of the human remains from the Farneto rock shelter hereby presented can confirm the existence of intentional cleaning practices and dismembering of corpses soon after death. While a particular predilection for crania has been often highlighted by literature so far (Miari 2013; Miari et al. 2017; Cavazzuti 2018; Cavazzuti et al. 2020; Belcastro et al. 2021), the careful study of the whole Farneto rock shelter skeletal remains assemblage also allowed to detect traces of disarticulation and scarnification not only on cranial districts, but also on postcranial bones.

In Neo/Eneolithic Europe, many sites have been excavated and various funerary behaviours documented, including primary and secondary depositions, single and collective burials (sometimes proper mass graves), both in open air spaces and caves or rock shelters (cf. Silva 2003; Köhler 2008; Bondár and Szécsényi-Nagy 2020; González-Rabanal et al. 2020). Besides single primary burials, the taphonomic study of the human remains led to hypothesise the presence of funerary treatment of corpses and modification of human bones, episodes of intergroup violence in the form of raids and ambushes and cannibalistic practices. For the Farneto rock shelter, we have not found evidence of extensive intergroup violence as documented for example at Schöneck-Kilianstädten (Germany, Neolithic LBK; Meyer et al. 2014, 2015), Talheim (Germany, Neolithic LBK; Meyer et al. 2014) and Potočani (Croatia, Eneolithic; Janković et al. 2021). In fact, we observed very few specimens, especially crania (Table 4; S2), with lesions compatible with violent strokes (even if the number could be underestimated due to the bad state of preservation of the bones).

As regards cannibalism, Bello et al. (2016) found that cut marks, indicative of defleshing or cleaning practices, are particularly frequent in cannibalised remains (more than 65% in Gough Cave, UK, Magdalenian), while they show very low frequencies in human bones treated for ritual or funerary purposes (below 1.5% in three Meso/Neolithic Serbian sites). As regards disarticulation traces, they are present in both persistent and labile joints in the case of cannibalism, while they are found especially on persistent joints when bones are ritually treated after a period of decomposition. In the Farneto rock shelter assemblage, the pattern observed is consistent with a funerary treatment of corpses, as already supposed from the interpretation of the ERI. In addition, strong evidence of alimentary cannibalism can be considered when, in the same site, human and animal remains are butchered with similar techniques and present similar patterns of long bone breakage aimed at marrow extraction, as in the case of the Neolithic site of Fontbrégoua (France; Villa et al. 1986). Convincing evidence of corpse exploitation to obtain nutrient substances comes also from Herxheim (Germany, Neolithic LBK; Boulestin et al. 2009). However, in this site, the good preservation of human remains allowed a detailed study of bone lesions and breakage patterns, not possible for the Farneto rock shelter assemblage. Moreover, the faunal remains in our sample are scarce and have never been analysed; thus, no hypothesis of cannibalism can be proposed on the basis of a comparison between the treatment of human and animal remains. It must be pointed out that cannibalism could also be accounted to a ritual funerary behaviour, without alimentary purposes, that would be even more difficult to detect and interpret (cf. Santana et al. 2019).

## Conclusions

Despite intrinsic difficulties, the present chronological and anthropological revision of the fragmented and commingled human remains from the Farneto rock shelter gave insights on many aspects that were previously unknown. These concern radiometric dating, the degree of fragmentation of the skeletal materials, the biological profile of the individuals and the taphonomic events in which they were involved.

The results of the radiometric and taphonomic analyses support for the first time an early use of natural cavities for funerary purposes in the central area of Emilia Romagna, starting from the final phase of the Neolithic period. Moreover, the assemblage represents one of the first documented cases in the region of perimortem intentional treatment of corpses, consisting of dismembering and scarnification. This has broadened our understanding of Neo/Eneolithic Italian funerary practices in the frame of the European context, where a variety of funerary behaviours and modes of corpse treatment and disposal are documented.

The present research highlights the importance of a careful restudy of human skeletal remains from prehistoric funerary contexts, especially aimed at detecting possible perimortem lesions that may have been underestimated or misinterpreted during previous studies. This would enrich the corpus of studies available for comparison in order to interpret other complex human skeletal assemblages, in particular those consisting of fragmented and commingled remains.

## Supplementary Information

Below is the link to the electronic supplementary material.

Supplementary file1 (PDF 115 KB)

Supplementary file2 (PDF 304 KB)

Supplementary file3 (PDF 166 KB)

Supplementary file4 (PDF 131 KB)

## Data Availability

All the information pertaining to the materials are available upon request directed to the corresponding author.
