# Supplementary material for: On the traces of lost identities: chronological, anthropological and taphonomic analyses of the Late Neolithic/Early Eneolithic fragmented and commingled human remains from the Farneto rock shelter (Bologna, northern Italy)
Source: Archaeol Anthropol Sci. 2023 Mar 1;15(3):36. doi: 10.1007/s12520-023-01727-2 (PMC9974402; doi:10.1007/s12520-023-01727-2)
Supplement: Supplementary file 1 — Supplementary file1 (PDF 115 KB) [file 12520_2023_1727_MOESM1_ESM.pdf]

On the traces of lost identities: chronological, anthropological and taphonomic analyses of the Late Neolithic/Early Eneolithic fragmented and commingled human remains from the Farneto rock shelter (Bologna, northern Italy)

Archaeological and Anthropological Sciences

Teresa Nicolosi[1,2], Valentina Mariotti[1], Sahra Talamo[3,4], Monica Miari[5], Laura Minarini[6], Gabriele Nenzioni[7], Fiamma Lenzi[7], Annalisa Pietrobelli[1], Rita Sorrentino[1,2], Stefano Benazzi[2], Maria Giovanna Belcastro[1]

[1]Department of Biological, Geological and Environmental Sciences, Alma Mater Studiorum University of Bologna, Bologna, Italy
[2]Department of Cultural Heritage, Alma Mater Studiorum University of Bologna, Bologna, Italy
[3]Department of Chemistry 'Giacomo Ciamician', Alma Mater Studiorum University of Bologna, Bologna, Italy
[4]Department of Human Evolution, Max Planck Institute for Evolutionary Anthropology, Leipzig, Germany
[5]Soprintendenza Archeologia, Belle Arti e Paesaggio per la città metropolitana di Bologna e le province di Modena, Reggio Emilia e Ferrara, Bologna, Italy
[6]Museo Civico Archeologico, Bologna, Italy
[7]Museo della Preistoria 'Luigi Donini', San Lazzaro di Savena, Bologna, Italy

Corresponding author
Maria Giovanna Belcastro mariagiovanna.belcastro@unibo.it

S1 Integration regarding the splitting of the materials from the Farneto rock shelter

During the long period of the discoveries, the findings from the Farneto rock shelter were brought to the 'Museo Civico Archeologico' of Bologna (MCA) and to the former Institute of Anthropology of the University of Bologna. In addition, some remains were previously kept by the 'Unione Speleologica Bolognese' and finally consigned to the University of Bologna (Facchini 1972). Moreover, some materials are stored at the 'Museo della Preistoria Luigi Donini' in San Lazzaro di Savena (MPLD).
The splitting of the materials among three different institutions derives from the relationships that L. Fantini had with the MCA, as an employee of the Municipality of Bologna, and with the speleological associations active in the exploration and study of the northern Apennines.
The major part of the material recovered was stored at the MCA, but the interest raised by the remains of the ancient inhabitants of the province of Bologna resulted in entrusting some of the human bones to the former Institute of Anthropology of the University of Bologna. In particular, the material recovered in 1935 was given to F. Frassetto, who had previously received the skeletal remains from the Farneto Cave (the cranial remains

of an adult female, an adult male and a child, together with some postcranial bones; Frassetto 1905). Subsequently, the skeletal remains were given to F. Facchini, who also participated in the recovery of the materials on some occasions (Facchini 1970).

As regards the MPLD, the archaeological materials and human remains that are part of the historical collections mostly derive from the explorations carried out during the 1950s by some associations which operated in the area of the 'Parco dei Gessi Bolognesi' (among these, the 'Pattuglia Archeologica Speleologica Scout', then 'Scientifica' - PASS, and the 'Centro Studi Archeologici' - CSA). Their recoveries and findings were originally stored at the so-called 'Antiquarium della Croara', refunded with the name of 'Museo della Preistoria' in 1985. Lastly, a small batch of materials was given to the museum by the 'Soprintendenza Archeologia, Belle Arti e Paesaggio per la città metropolitana di Bologna e le province di Modena, Reggio Emilia e Ferrara' (SABAP-BO), including findings almost certainly brought by L. Fantini himself.

References

Facchini F (1970) Nuovi rinvenimenti scheletrici nei pressi della grotta del Farneto (Bologna). Nota preventiva. Tipografia "La Grafica Emiliana", Bologna

Facchini F (1972) I reperti osteologici della stazione del Farneto e il loro interesse antropologico. Estratto da Memoria X della Rassegna Speleologica Italiana. Atti del VII Convegno Speleologico dell'Emilia-Romagna e del Simposio di studi sulla Grotta del Farneto

Frassetto F (1905) Frammenti di scheletri umani rinvenuti nella grotta del Farneto presso Bologna. Proteus, 3, f. II-III, 1–6
