# Supplementary material for: On the traces of lost identities: chronological, anthropological and taphonomic analyses of the Late Neolithic/Early Eneolithic fragmented and commingled human remains from the Farneto rock shelter (Bologna, northern Italy)
Source: Archaeol Anthropol Sci. 2023 Mar 1;15(3):36. doi: 10.1007/s12520-023-01727-2 (PMC9974402; doi:10.1007/s12520-023-01727-2)
Supplement: Supplementary file 2 — Supplementary file2 (PDF 304 KB) [file 12520_2023_1727_MOESM2_ESM.pdf]

On the traces of lost identities: chronological, anthropological and taphonomic analyses of the Late Neolithic/Early Eneolithic fragmented and commingled human remains from the Farneto rock shelter (Bologna, northern Italy)

Archaeological and Anthropological Sciences

Teresa Nicolosi[1,2], Valentina Mariotti[1], Sahra Talamo[3,4], Monica Miari[5], Laura Minarini[6], Gabriele Nenzioni[7], Fiamma Lenzi[7], Annalisa Pietrobelli[1], Rita Sorrentino[1,2], Stefano Benazzi[2], Maria Giovanna Belcastro[1]

[1]Department of Biological, Geological and Environmental Sciences, Alma Mater Studiorum University of Bologna, Bologna, Italy

[2]Department of Cultural Heritage, Alma Mater Studiorum University of Bologna, Bologna, Italy

[3]Department of Chemistry 'Giacomo Ciamician', Alma Mater Studiorum University of Bologna, Bologna, Italy

[4]Department of Human Evolution, Max Planck Institute for Evolutionary Anthropology, Leipzig, Germany

[5]Soprintendenza Archeologia, Belle Arti e Paesaggio per la città metropolitana di Bologna e le province di Modena, Reggio Emilia e Ferrara, Bologna, Italy

[6]Museo Civico Archeologico, Bologna, Italy

[7]Museo della Preistoria 'Luigi Donini', San Lazzaro di Savena, Bologna, Italy

Corresponding author

Maria Giovanna Belcastro mariagiovanna.belcastro@unibo.it

S2 Perimortem lesions detected on postcranial bones with the indication of district, ID, type of bone and state of preservation, side, sex and age of the individual, type and position of the lesion, description and interpretation distinguished for sharp force trauma and fractures. State of preservation is indicated following the standard described in 'Methods' section. Abbreviations: SP, state of preservation; NR, not recordable; M, male; F, female; AD, adult; YA, young adult; MA, mature adult; SUB, subadult; CH, child; ADOL, adolescent

District	ID	Bone (SP)	Side	Sex, Age	Sharp force lesions				Fractures	
					Type	Position	Description	Interpretation	Description	Interpretation
**Vertebrae**	C5-BL2-SN	Lumbar vertebra (5.3)	-	AD	Cut or chop mark with crushing	Anterior margin of the superior surface of the body	The lesion (11x2 mm) is deeper on the anterior margin; on the left margin the cortical is interrupted, while on the right side the surface is continuous and sloping.	Disarticulation.		
	C5-BL3-SN	Atlas (2)	-	AD	Cut mark and breakage?	Right massa lateralis	Fracture involving the whole thickness of the bone; on the condylar surface the characteristics of the lesion are not recordable because it is completely filled with sediments and proper margins of the fracture are not visible; on the inferior articular surface, the lateral portion of the lesion presents straight margins (cut mark?), while the medial portion shows a crushed area with several adhering flakes.	Disarticulation of the cranium from the column.		
	C5-BL3-SN	Cervical vertebra (3rd?) (5.3)	-	AD	Cut marks	Left superior articular surface	Two cut marks, departing from the posterior part of the superior articular surface, reach the posterior part of the articular process; the end of the lesions cannot be detected because the bone is broken on its inferior part; the distance between the two cut marks is 4 mm; their overall length reaches around 10 mm (Fig. 8a, b).	Disarticulation of the cranium from the column.		
**Ribs**	C1-AMH-269	Rib (2nd?) (5.3)	right	AD	Crushing lesion	Superior surface, anteriorly to the tubercle	The crushing lesion (12x2 mm) shows a U-shaped bottom with transversal rounded fractures; the posterior margin is straighter and steeper; along the anterior margin, the cortical is interrupted (Fig. 9a). In addition, along the anterior margin, an incomplete fracture line delimitates a slightly depressed area (9x3 mm).	Cleaning practices or dismembering.		
	C1-AMH-270	Rib (5.3)	NR	AD	Cut mark	Inner surface of the body	The thin linear cut mark (18 mm) is interrupted on one side because the bone is broken; it shows internal parallel microstriae and straight regular margins; flaking on one side is very slightly visible (Fig. 8c).	Cleaning practices or dismembering.		
	C5-BL1-SN	Rib (5.3)	left?	AD	Chop mark	Superior margin of the body on the external surface	The chop mark (4x4 mm, depth 1 mm) shows an almost symmetrical V-shape with a small breakage on the anterior margin. Anteriorly to the chop mark, a possible thin cut mark (4 mm) is visible.	Cleaning practices or dismembering.		
	C5-BL1-SN	Rib (5.3)	NR	AD	Crushing lesion	Inferior margin of the body on the external surface	The lesion (14x3 mm) has an oblique orientation; its superior extremity is shallower and the cortical is there continuous; in the remaining part, the lesion has caused cortical breakage. Inferiorly, an adhering flake is delimited by an incomplete fracture.	Cleaning practices or dismembering.		
**Shoulder girdle**	C5-BL1-52	Scapula (5.3)	left	CH	Chop mark with crushing	Inferior part of the spine	The crushing lesion (6x2 mm) has a V-shape; the crushed surface is delimitated by a thin line of fracture along the lateral margin.	Disarticulation of the shoulder joint.		
**Upper limb**	C7-AMH-232	Humerus (4)	right	AD	Cut marks	Posterior proximal third and lateral distal third of the diaphysis	At least three transversal thin cut marks (around 5 mm) are visible on the posterior diaphyseal surface (Fig. 8d, e). Among these, the most distal one shows flaking on the inferior margin and traces of sediment on the bottom (Fig. 8e). Another oblique cut mark (10 mm) is visible on the lateral distal third of the diaphysis; its distal margin is more sloping than the proximal one. Another possible lesion of triangular shape (2 mm) is located laterally to the most distal of the three thin cut marks.	Defleshing.		

	C7-AMH-236	Humerus (5.2)	right	SUB	Cut mark	Posterior distal third of the diaphysis	The cut mark (10 mm) is transversally oriented, with an almost regular V-shaped bottom; parallel microstriae on the lateral end (Fig. 8f).	Defleshing.		
	C4-58	Humerus (4)	right	MA	Chop marks	Posterior distal diaphysis and epiphysis	The most proximal chop mark (10x6 mm, but a small fragment of bone may have been lost from the inferior margin) on the posterior medial surface of the diaphysis shows a V-shaped bottom with a very thin apex. Below this, another possible chop mark (8x7 mm) is visible. Another chop mark (8x3 mm) is transversally oriented on the posterior part of the lateral epicondyle; the superior margin is steeper, while the inferior one is more sloping; some sediments fill the most lateral part of the lesion, while on the medial part some longitudinal internal striae are visible. Another long chop mark (40 mm) crosses the posterior surface of the distal epiphysis, tangent to the medial border of the olecranon fossa, with the extremities filled with some sediments; the overall width is not detectable because part of the epiphysis is broken.	Powerful or violent dismembering (interpersonal or ritual violence?).	The breakage at the mid-diaphysis presents some characteristics of a perimortem fracture on its lateral part, where a flake of cortical bone with a parabolic outline has been detached, leaving an irregular surface.	The overall features are more compatible with dry or mineralised bone fractures, but we cannot exclude some damage occurred during the practices of treatment of the corpse that also caused the chop marks.
	C5-BL1-SN	Humerus (3)	right	AD	Chop mark	Anterior proximal diaphysis	The chop mark (8x1 mm) is located transversally to the latissimus dorsi and teres major enthesis; it has a V-shaped bottom with thin apex; the inferior margin is more irregular than the superior one.	Defleshing and/or disarticulation.		
	C5-BL1-SN	Ulna (3)	right	CH	Puncture wound	Medial proximal diaphysis	The lesion (5x3 mm) has an oval shape likely produced by the tip of a blade. A small rectangular flake (4x1 mm) has detached from the posterior margin.	The lesion could be accidentally produced during other practices of treatment of the corpse.		
**Pelvic girdle**	C8-114	Ischium (5.3)	right	AD	Chop mark	Anterior part of the ramus below the acetabular cavity	The V-shaped chop mark (6x2 mm) is obliquely oriented, with superior margin more regular and inferior one with some flaking (Fig. 9b).	Disarticulation of the hip joint.		
**Lower limb**	C4-AMH-298	Femur (5.2)	right	AD	Cut mark and perforating lesion	Proximal third of the linea aspera	The cut mark (10 mm) is obliquely oriented on the distal end of the gluteus maximus enthesis, with several parallel microstriae on the bottom. The perforating lesion (14x4 mm) of elliptical shape is located below the intertrochanteric line; it shows regular margins, not completely preserved. A flake of cortical bone (14x8 mm) of irregular shape has been detached 12 mm below the perforating lesion; its superior part is straight and nearly perpendicular.	Disarticulation or dismembering and/or defleshing. Probably violent actions caused the perforating lesion (interpersonal or ritual violence?).	The proximal bone breakage has a helical fracture outline, but the surface is quite irregular. The distal fracture presents a mixed outline (linear and curved) and an irregular surface, perpendicular in about one third of the circumference. Spalling of cortical bone (15x12 mm) is visible on its lateral part, with sediments adhering to the rupture surface.	Probably dry bone breakage, even if some perimortem damages occurred during the practices of treatment of the corpse cannot be excluded.
	C4-AMH-298	Femur (5.2)	left?	AD	Cut mark	Proximal third of the diaphysis	A possible cut mark is visible on the posterior medial surface near to the proximal breakage, but the state of preservation is bad due to cortical exfoliation.	Defleshing.	At least one portion of the proximal bone breakage presents an acute angle between fracture and bone surfaces.	Probably dry bone breakage, even if perimortem damage cannot be excluded.
	C5-AMH-79	Femur (5.2)	left	AD	Cut mark	Distal end of the linea aspera	The fragment includes the most distal extremity of the linea aspera and the lateral supracondylar line; the thin cut mark (6 mm) is obliquely oriented laterally to the linea aspera.	Defleshing.	The bone flake shows an oval profile, smooth fracture surfaces and acute proximal fracture angle (Fig. 10e).	The whole bone flake may be the result of a fresh bone breakage, possibly procured during treatment of the corpse.
	V-AMH-211	Femur (2*)	left	F, YA	Chop marks and crushing lesions	Anterior aspect of the femoral head	A V-shaped chop mark (20x6 mm) crosses the femoral head epiphyseal line obliquely; in the two extremities of the chop mark the cortical is crushed (in the medial part the cancellous bone is exposed probably due to	Powerful or violent disarticulation or dismembering (interpersonal or ritual violence?; cf.	The transversal fracture at the proximal third of the diaphysis was probably created postmortem and glued during	Treatment of the corpse could have caused the spalling and an incomplete fracture that then completely broke postmortem.

						and lesser trochanter	postmortem damage). Another chop mark, that caused the detachment of the proximal extremity (including head, neck and the superior portion of the greater trochanter, glued during previous studies), was probably inflicted from the posterior surface of the neck; a triangular portion (28x8 mm) of bone from the base of the neck has been lost. The third chop mark (10x1 mm) is obliquely oriented on the lesser trochanter (enthesis of the iliopsoas muscle), with a V-shaped bottom (Fig. 9c). At least three crushing lesions are detectable on the femoral head; one is circular (6 mm), one is elliptical (10x5 mm), while the last one is irregular in shape (9x6 mm).	humerus C4-58 and femur C4-AMH-298).	previous studies, with spalling of cortical bone on the proximal (14x18 mm) and distal (6x5 mm) fragments in correspondence of the linea aspera.	
	V-AMH-212	Femur (2*)	left	F, YA	Chop, cut, scrape marks and crushing lesions	Femoral condyles and diaphysis	The chop mark (9x4 mm) on the posterior surface of the lateral condyle is asymmetric. Three cut marks (maximum length 6 mm) are present on its medial extremity (the lateral one is not recordable because of postmortem damage). Other two thin curvilinear cut (?) marks (around 9 mm) are visible on the anterior part of the medial condyle. A crushed lesion (13x10 mm) of irregular shape is located on the inferior surface of the medial condyle. Other crushed lesions are visible on the inferior medial portion of the lateral condyle, one resembling a chop mark (13x3 mm) and the other one of irregular shape (12x4 mm). An elliptical and elongated crushing lesion or chop mark (16x4 mm) on the lateral aspect of the distal third of the diaphysis has a U-shaped bottom. About 90 mm above this lesion, a flake (17x9 mm) of cortical bone of irregular shape has been detached. Scrape marks (around 30 mm) with removal of a thin strip of cortical bone are visible on the proximal extremity of the gluteus maximus enthesis.	Disarticulation and defleshing. The curvilinear marks could be due to trampling as well.	Two fractures were glued during previous studies, but present a spiral profile, typical of fresh bone. The proximal one involves the femoral neck, obliquely broken, with the blow probably originating from the inferior portion of the neck (cf. femur V-AMH-211). The distal fracture is located at the distal third of the diaphysis. Two incomplete fracture lines are visible along the greater and lesser trochanter.	Dismembering?
	V-AMH-213	Femur (5.2)	right	M, AD	Cut mark	Proximal third of the medial surface of the diaphysis	The thin cut mark (4 mm) is obliquely oriented.	Cleaning practices.	The broken extremities of the diaphysis present features of dry and mineralised bone fractures. Spalling of the cortical bone is present at both extremities (more in detail, a triangular area of 20x16 mm at the proximal one).	Probably mineralised bone breakage, even if perimortem damage cannot be excluded.
	C5-BL1-SN	Femur (4)	left	AD	Chop mark	Lateral condyle	A deep V-shaped chop mark (16x2 mm) is almost vertically oriented on the medial portion of the lateral condyle. Adjacent to its lateral margin, a second lesion (5 mm) is visible inferiorly.	Disarticulation.		
	C5-BL1-SN	Femur (5.2)	NR	AD	Chop mark	Diaphysis	A possible chop mark is located on the margin of a likely fresh bone fracture with a spiral profile.	Dismembering.	Spiral fractures with spalling of cortical bone are present at both extremities of the fragment, more evident on one end (Fig. 10c). One of these spalling areas is covered by sediments (Fig. 10d).	Probable dismembering practices.
	C5-BL2-SN	Femur (5.2)	right	AD	Chop mark	Anterior part of the medial surface of the diaphysis	The deep chop mark (18 mm) is obliquely oriented; its inferior margin is very regular and steep, while the superior one presents a partially detached big flake of bone (Fig. 9d).	Attempt to dismember or deflesh the bone with thick muscular masses.	The proximal bone breakage is very badly preserved because of weathering, but a portion of spiral fracture is visible.	Dismembering practices may have caused bone breakage.
	C12-79G	Femur (5.2)	right	AD	Chop and scrape marks	Proximal diaphysis	The V-shaped chop mark (12x10 mm) is located on the medial surface, below the femoral neck, with the inferior margin more regular than the superior one; some sediments fill the lesion. On the margin of the distal breakage, on the postero-lateral aspect, two	Disarticulation and dismemberment.	The proximal breakage presents incomplete fractures, spalling and compression of cancellous bone. The distal breakage shows	The proximal breakage may be at least partly related to perimortem activities, while the distal one was likely produced postmortem on a previously

							smooth contiguous areas related to a cutting surface (12x4 mm) and to a scraping surface (5x4 mm, a depressed surface with parallel striae) are visible.		mineralised bone features, with rough surface.	treated area (cf. femur V-AMH-211).
	C12-SN	Femur (5.2)	left	AD	Chop mark	Inferiorly to the greater trochanter	The chop mark (11x6 mm) is almost transversally oriented on the anterior surface, inferiorly to the greater trochanter; it has an irregular V-shape, with the superior margin very irregular and the inferior one particularly sloping.	Dismembering and/or defleshing.		
	C12-139	Femur (5.2)	right	M, AD	Chop and scrape marks	Distal diaphysis	The most distal lesion is a small shallow chop mark (7x1 mm). Superiorly, the biggest lesion (24x6 mm) is composed by two curvilinear sulci showing several parallel striae on the bottom. Another similar lesion (7x3 mm) is located superiorly to the previous one. More proximally, medially to the linea aspera, there is a scraped area (12x7 mm) with microstriae on the bottom. Adjacent to this, another similar area (around 25 mm) on the linea aspera is visible. Two other possible chop marks (16x7 mm and 9x7 mm) with internal microstriae and irregular surface of the sloping margins are present.	Defleshing. As regards the curvilinear sulci, rodent gnawing cannot be excluded.	The proximal breakage at about mid-diaphysis shows mixed features (a portion of transversal fracture with perpendicular surface, other portions with acute angle between fracture surface and bone surface), along with areas of peeling.	Perimortem interventions and subsequent dry bone damage?
	C12-137	Femur (2)	right	F, YA	Cut marks	Distal diaphysis	A possible longitudinal cut mark (25 mm) is located on the lateral diaphysis, reaching the distal breakage. Two possible transversal cut marks (maximum length 6 mm) are present on the postero-lateral aspect, near to the distal breakage.	Disarticulation or dismembering of the knee joint. The femur is almost complete and well preserved, but missing only the distal extremity.	An incomplete and depressed fracture (16x3 mm) is present along the medial aspect of the distal breakage.	Possible disarticulation or dismembering of the knee joint.
	MPLD-SN	Femur (3)	right	F?, YA	Cut mark	Medial surface of the mid-diaphysis	The oblique cut mark (10 mm) is located in correspondence of a slightly flattened area, whose bottom shows parallel striae.	Defleshing.		
	C7-AMH-246	Patella (1*)	right	AD	Cut marks	Medial articular surface	The longest thin oblique linear cut mark (13 mm) presents several longitudinal microstriae on the bottom (Fig. 8g). Inferiorly, at a distance of 1 mm, another cut mark (3 mm) is present. On the superior part of the surface, two thin cut marks (maximum length 7 mm) form a X-shape (Fig. 8h).	Careful cleaning purposes on an already disarticulated bone.		
	C8-AMH-279	Patella (1*)	left	AD	Penetrating lesion	Anterior surface	The linear penetrating wound (20 mm) is transversally oriented in the middle of the anterior surface, causing breakage of the bone in two parts glued during previous studies. Inferiorly to the lesion, a roughly rounded depressed area (20x12 mm) is delimitated by a superficial line of fracture.	Powerful dismembering, with blow inflicted from below (interpersonal or ritual violence?).		
	C5-BL3-140	Patella (1)	left	AD?	Chop mark	Anterior surface	The V-shaped chop mark (8x3 mm) presents irregular margins.	Dismembering or defleshing.		
	C4-AMH-298	Tibia (5.2)	left	AD	Cut, chop and scrape marks	Diaphysis	The most proximal lesion is represented by a circular depressed area (9 mm) with rough surface and at least two cut marks inside it, with parallel microstriae. Two opposed lesions are present at the mid-diaphysis; the anterior one (10x5 mm on the crest, extending laterally for other 7 mm) shows crushed and raised cortical bone; the posterior one (11x5 mm) is a chop mark with signs of scraping on the inferior border and an incomplete fracture below it.	Defleshing. The gastrocnemius muscle was probably chopped with the limb resting on its anterior portion, which shows an anvil scar.	Fractures at the two extremities present mixed features. On the proximal one, the latero-anterior surface shows a peeled area (30x12 mm), with a roughened exfoliated surface (Fig. 10f), associated with a possible chop mark on the anterior crest.	The chop mark could indicate dismembering practices, which also caused the peeling.
	C7-AMH-223	Tibia (1*)	right	M?, ADOL	Chop and scrape marks?	Diaphysis and articular	Two adjacent chop marks (14x4 mm and 8x2 mm) show very irregular surfaces and microstriae in some areas, or maybe a unique scraped area, on the medial surface of	Disarticulation of the ankle joint and defleshing. As regards the proximal lesion,	Butterfly fracture (glued during previous studies) at the distal third of the diaphysis (Fig. 10a),	Typical fresh bone fracture of intentional or accidental origin.

						surface of the malleolus	the proximal third of the diaphysis; the inferior margin is more regular with flaking. Another chop mark (8x1 mm) is present on the articular surface at the base of the malleolus; the distal part of the chop mark is filled with sediments. Posteriorly, another chop mark (3x1 mm) could be the prosecution of the same lesion or the result of a second blow.	rodent gnawing cannot be excluded.	from which an incomplete fracture line departs (Fig. 10b).	
	C5-BL1-SN	Tibia (5.2)	left	AD	Cut marks	Posterior medial diaphysis	Two thin parallel and oblique cut marks (8 mm and 5 mm) distant 3 mm from each other are visible. In this area, a flake of bone has been detached recently, as testified by the white colour.	Defleshing.		
	C5-AMH-83	Fibula (5.2)	left	AD	Cut mark	Medial diaphysis close to the nutrient foramen	The cut mark (5 mm) is obliquely oriented, with more regular and steeper inferior margin with flaking. A thin fracture line surrounds the superior margin.	Defleshing.	The fractures (partially glued during previous studies) appear produced on mineralised bone, but distally a fresh bone fracture with spiral profile and crushing of the margin is partially visible.	Dismembering?
	C7-AMH-231	Fibula (5.2)	left	AD	Cut mark	Lateral aspect of the distal third of the diaphysis	The thin cut mark (13 mm) is obliquely oriented.	Defleshing.		
	C4-159	Calcaneus (1)	right	CH	Penetrating lesion	Laterally to the posterior articular surface	The lesion (17x2 mm) is represented by a linear and deep (7 mm) penetrating wound (Fig. 9e).	Dismembering.		
	C4-166	Talus (1)	left	AD	Penetrating lesion?	Laterally to the calcaneal anterior articular surface	The lesion (17x2 mm) is represented by a linear and deep (6 mm) penetrating wound with very vertical margins (Fig. 9f).	Dismembering.		

*Symbol refers to elements restored during previous studies
