# Supplementary material for: On the traces of lost identities: chronological, anthropological and taphonomic analyses of the Late Neolithic/Early Eneolithic fragmented and commingled human remains from the Farneto rock shelter (Bologna, northern Italy)
Source: Archaeol Anthropol Sci. 2023 Mar 1;15(3):36. doi: 10.1007/s12520-023-01727-2 (PMC9974402; doi:10.1007/s12520-023-01727-2)
Supplement: Supplementary file 3 — Supplementary file3 (PDF 166 KB) [file 12520_2023_1727_MOESM3_ESM.pdf]

On the traces of lost identities: chronological, anthropological and taphonomic analyses of the Late Neolithic/Early Eneolithic fragmented and commingled human remains from the Farneto rock shelter (Bologna, northern Italy)

Archaeological and Anthropological Sciences

Teresa Nicolosi[1,2], Valentina Mariotti[1], Sahra Talamo[3,4], Monica Miari[5], Laura Minarini[6], Gabriele Nenzioni[7], Fiamma Lenzi[7], Annalisa Pietrobelli[1], Rita Sorrentino[1,2], Stefano Benazzi[2], Maria Giovanna Belcastro[1]

[1]Department of Biological, Geological and Environmental Sciences, Alma Mater Studiorum University of Bologna, Bologna, Italy

[2]Department of Cultural Heritage, Alma Mater Studiorum University of Bologna, Bologna, Italy

[3]Department of Chemistry 'Giacomo Ciamician', Alma Mater Studiorum University of Bologna, Bologna, Italy

[4]Department of Human Evolution, Max Planck Institute for Evolutionary Anthropology, Leipzig, Germany

[5]Soprintendenza Archeologia, Belle Arti e Paesaggio per la città metropolitana di Bologna e le province di Modena, Reggio Emilia e Ferrara, Bologna, Italy

[6]Museo Civico Archeologico, Bologna, Italy

[7]Museo della Preistoria 'Luigi Donini', San Lazzaro di Savena, Bologna, Italy

Corresponding author

Maria Giovanna Belcastro mariagiovanna.belcastro@unibo.it

S3 Comparison of the element representation index (ERI) from the Farneto rock shelter and other archaeological contexts (data from Knüsel et al. 2016): Scaloria Cave (Foggia, Apulia, southern Italy, Neolithic; corpse manipulation and secondary burials), Kunji Cave (Iran, Bronze Age; collective and secondary burials with probable bone selection) and West Tenter Street (London, UK, Roman period; single primary burials). Abbreviations: H, hand; F, foot

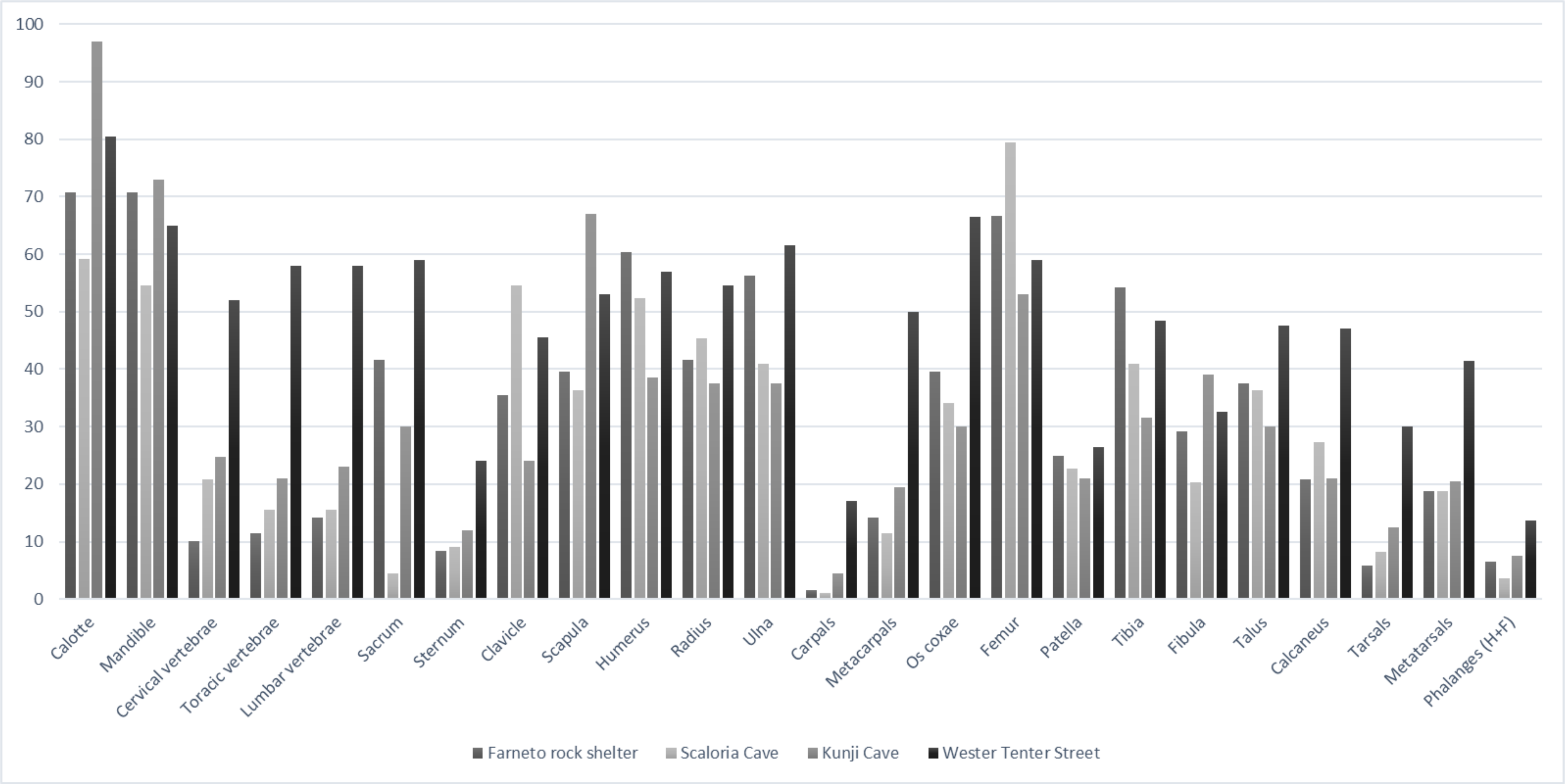

References

Knüsel C, Robb J, Tafuri MA (2016) The Upper Cave: Taphonomic Analysis of the Treatment of the Dead. In: Elster ES, Isetti E, Traverso A, Robb J (eds) The Archaeology of Grotta Scaloria. Ritual in Neolithic Southeast Italy. Monumenta Archaeologica 38:145–191
